# Localisation and origin of the bacteriochlorophyll-derived photosensitizer in the retina of the deep-sea dragon fish *Malacosteus niger*

**DOI:** 10.1038/srep39395

**Published:** 2016-12-20

**Authors:** Ronald H. Douglas, Martin J. Genner, Alan G. Hudson, Julian C. Partridge, Hans-Joachim Wagner

**Affiliations:** 1 Division of Optometry & Visual Science, City, University of London, Northampton Square, London EC1V 0HB, UK; 2 School of Biological Sciences, University of Bristol, Bristol Life Sciences Building, 24 Tyndall Avenue, Bristol BS8 1TQ, UK; 3 Oceans Institute, School of Animal Biology, M092, The University of Western Australia, 35 Stirling Highway, Crawley WA 6009, Australia; 4 Anatomisches Institut der Universität Tübingen, Ősterbergstrasse 3, 72074 Tübingen, Germany

## Abstract

Most deep-sea fish have a single visual pigment maximally sensitive at short wavelengths, approximately matching the spectrum of both downwelling sunlight and bioluminescence. However, *Malcosteus niger* produces far-red bioluminescence and its longwave retinal sensitivity is enhanced by red-shifted visual pigments, a longwave reflecting tapetum and, uniquely, a bacteriochlorophyll-derived photosensitizer. The origin of the photosensitizer, however, remains unclear. We investigated whether the bacteriochlorophyll was produced by endosymbiotic bacteria within unusual structures adjacent to the photoreceptors that had previously been described in this species. However, microscopy, elemental analysis and SYTOX green staining provided no evidence for such localised retinal bacteria, instead the photosensitizer was shown to be distributed throughout the retina. Furthermore, comparison of mRNA from the retina of *Malacosteus* to that of the closely related *Pachystomias microdon* (which does not contain a bacterichlorophyll-derived photosensitzer) revealed no genes of bacterial origin that were specifically up-regulated in *Malacosteus*. Instead up-regulated *Malacosteus* genes were associated with photosensitivity and may relate to its unique visual ecology and the chlorophyll-based visual system. We also suggest that the unusual longwave-reflecting, astaxanthin-based, tapetum of *Malacosteus* may protect the retina from the potential cytotoxicity of such a system.

As both downwelling sunlight and bioluminescence in the deep-ocean are spectrally restricted (centred around 470–490 nm), the vast majority of deep-sea fish have visual systems maximally sensitive in this part of the spectrum^1^,^2^. An exception are three genera of stomiid dragon fish, that not only produce longwave bioluminescence^3^,^4^,^5^ but also have longwave-shifted visual pigments compared to other deep-sea animals^1^,^6^,^7^,^8^,^9^,^10^,^11^,^12^,^13^. This ‘red-shift’ in bioluminescence and visual sensitivity provides these animals with a ‘private waveband’ that might be used for both covert illumination of prey and interspecific signalling immune from detection by potential predators. In one species, *Malacosteus niger*, longwave sensitivity is, uniquely, further enhanced by a bacteriochlorophyll c&d-derived photosensitizer that absorbs illumination strongly around 675 nm^9^,^14^,^15^. Long wavelength sensitivity in this species is additionally increased by an astaxanthin-based retinal tapetum that reflects red light for a second pass through the photoreceptor layer^1^,^8^,^9^,^16^.

Photosensitization in *Malacosteus* is poorly understood. One of the many outstanding questions is the origin of the bacteriochlorophyll c&d, the best known sources of which are photosynthetic green sulphur bacteria of the family Chlorobiaceae and green non-sulphur bacteria of the family Choroflexaceae. We have previously suggested that the photosensitizer may reach the retina via a food chain involving copepods^17^. However, an interesting alternative is that the bacteria producing the bacteriochlorophyll are endosymbiotic in the retinae of *Malacosteus*. Although, this possibility might at first sight appear remote, endosymbiotic bacteria fulfil many functions in vertebrates, including the production of bioluminescence in many deep sea fish^18^. Such bacterially mediated bioluminescent symbiosis has evolved at least 27 times across diverse orders of ray-finned fishes^19^, indicating the scope for the evolution of such prokaryote/eukaryote relationships. A third, less likely, possibility is that the photosensitizer is manufactured by *Malacosteus* itself with appropriate genes being incorporated into the animal’s genome, potentially by horizontal gene transfer from prokaryotes.

The morphology of the *Malacosteus* retina has been examined previously^8^,^9^,^20^. Interestingly, Brauer^20^ described an unusual layer of structures between the retinal pigment epithelium (RPE) and the rod outer segments (see Supplementary File S1). Since these retinal structures appear to be unique to *Malacosteus*, the only species proven to contain a bacteriochlorophyll-derived retinal photosensitizer, and as they are adjacent to the photoreceptor outer segments where the bacteriochlorophyll has its physiological effect, it appears possible they may in some way be involved with bacteriochlorophyll storage/production. They might, for example, be the location of endosymbiotic bacteria.

Here we examine the structure of the *Malacosteus* retina by both light and electron microscopy, paying particular attention to the unusual structures described by Brauer^20^. We also characterise the molecular nature of the outer retina by electron energy loss spectroscopy (TEM EELS) to understand its elemental profile, and use SYTOX green staining to locate nucleic acids of eukaryotic or prokaryotic origin. The distribution of the bacteriochlorophyll-derived photosensitizer throughout the retina is investigated by absorption and fluorescence spectroscopy of cryosections. Finally, mRNA isolated from the *Malacosteus* retina is compared to that of a close relative, *Pachystomias microdon*, to identify genes that are differentially upregulated in *Malacosteus*. Since the *Malacosteus* retina is known to contain a photosensitizer while that of *Pachystomias* does not, any signal unique to the former might be related to photosensitization.

## Results

### Light & electron microscopy

**Figure 1:**
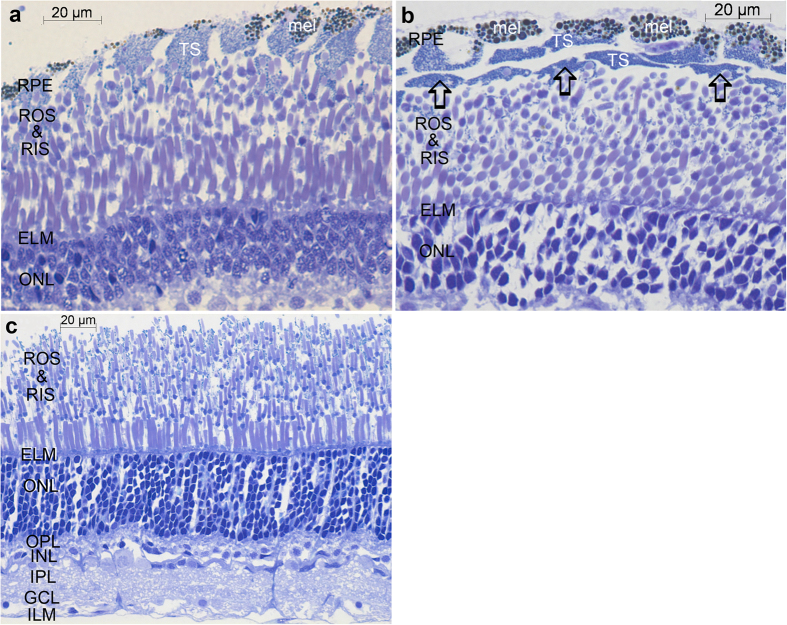
Transverse toluidine blue stained light microscopic sections of the *Malacosteus niger* retina. (**a**,**b**) show sections of outer retina taken from peripheral regions encompassing the outer nuclear layer (ONL) to the retinal pigment epithelium (RPE). Rod outer segments (ROS) and more darkly stained inner segments (RIS) form several superimposed tiers. Section (**a**) is cut relatively radially and the RPE cells appear as a single columnar layer with brown melanin pigmentation (mel) sclerally and lighter tapetal spheres (TS) vitreal to the melanin. In (**b**) the section is cut more obliquely and the RPE thus appears to form 2–3 discrete layers. The arrows indicate oblique sections through the vitreal region of the RPE cells containing the tapetal spheres which, due to the plane of section, appear to be separate from the more scleral part of the RPE cell. ELM–external limiting membrane. (**c**) is a section of the entire retina taken from more central regions. Here, unlike in the peripheral retina, the photoreceptors nearest the external limiting membrane (ELM) have larger outer segments (ROS) than more scleral rods. There are also more tiers of photoreceptors than peripherally. OPL–outer plexiform layer; INL–inner nuclear layer; IPL–inner plexiform layer; GCL–ganglion cell layer; ILM–internal limiting membrane. In this section the retinal pigment epithelium has become detached.

In agreement with previous work^8^,^9^, the photoreceptor layer of the *Malacosteus* retina is made up of several tiers of rod inner and outer segments (RIS/ROS): a regular layer near the external limiting membrane (ELM) and more disorganised scleral layers in which the outer and inner segments lie at various distances from the ELM (Fig. 1). In the central retina the rods nearest the ELM have longer (19–22 μm) and larger diameter (2.5 μm) outer segments than those situated more sclerally (Fig. 1c). Elsewhere in the retina the ROS in all layers are comparatively small (typically 8–10 μm long and barely 2 μm wide; Fig. 1a).

**Figure 2:**
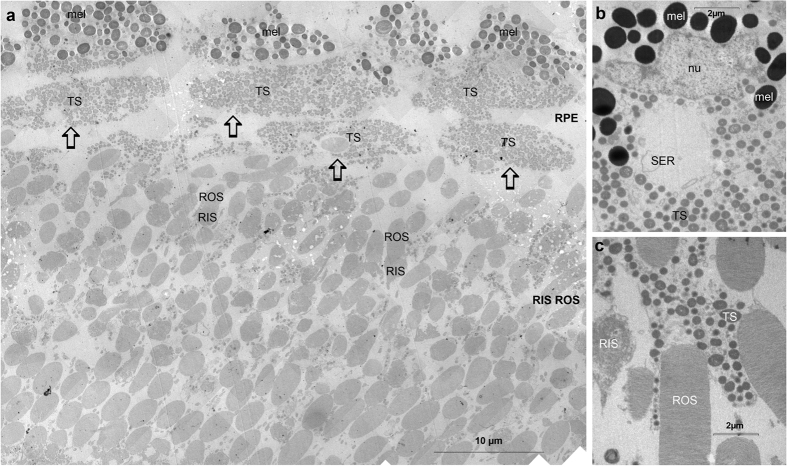
Electron micrographs of the *Malacosteus niger* retina. (**a**) Low power electron micrograph of the obliquely sectioned area shown in Fig. 1b. (**b**) shows the perinuclear region of a radially sectioned RPE cell. The nucleus (nu) is surrounded sclerally by darker melanosomes (mel; diameter up to 2 μm) and vitrally by numerous smaller (0.5-1 μm) tapetal spheres (TS) as well as smooth endoplasmic reticulum (SER). (**c**) demonstrates the microvillous-like projections of radially sectioned RPE cells which interdigitate with rod outer segments (ROS) and are filled with tapetal spheres which are much less electron dense than the melanosomes and match the lipid/membrane-rich ROS in density. The melanosomes in (a) appear lighter than those in (b) as only the latter are stained with lead citrate. All other labels as in Fig. 1

**Figure 3:**
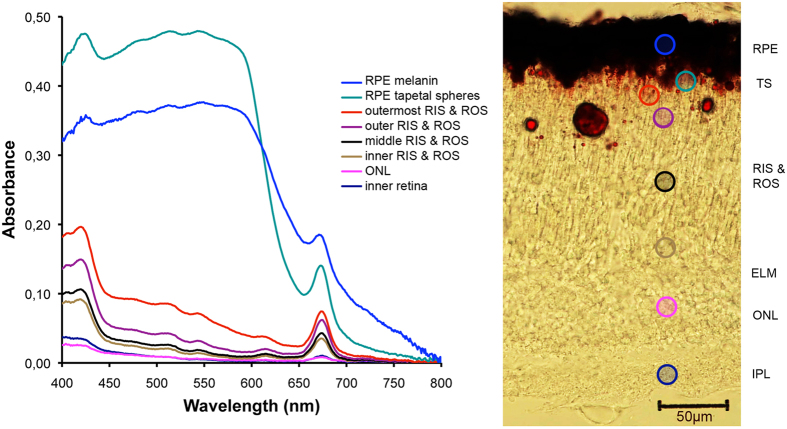
Unstained cryosection of a *Malacosteus niger* retina with associated spectral absorption scans from different layers. Each curve is an average of scans from 5 different retinal locations at eight different levels in each retina. All scans have been zeroed at 800 nm. The shape of these absorption spectra has characteristics of a chlorophyll-like pigment with absorption peaks close to those observed in extracts of BChl (see Supplementary Fig. S4). The area between the brownish melanin-rich scleral region of the RPE and the RIS/ROS appears bright red and corresponds to the astaxanthin-containing tapetal spheres of the vitreal RPE. Some tapetal spheres have become dislocated to the RIS/ROS layer during cryosectioning. All labels as in Fig. 1.

The RPE cells of most deep-sea fish are poorly developed and squamous^21^,^22^,^23^. Unusually, those of *M. niger* are relatively well developed and columnar^8^,^9^ (Fig. 1a). Sclerally they contain unusually large (up to 2 μm) dark melanin granules, while more vitreally they are filled with smaller inclusions which represent the astaxanthin-containing tapetal spheres (Figs 1 and 2). As the astaxanthin will have dissolved during histological processing, these structures appear colourless in conventional light and electron micrographs. Their true red colouration is, however, apparent in cryosections (Fig. 3) in which it is seen most clearly at the vitread margin of the RPE where it acts as a tapetal reflector.

We were most interested in characterising the unusual layer identified by Brauer^20^ between the RPE cells and the rods (see Supplementary File S1). He noted these structures had the same width as the RPE cells and were therefore probably derived from them and most likely represented the tapetum. In a sense he was correct, although we do not think they represent a distinct retinal layer. We believe they are instead an artefact caused by the plane of sectioning and the columnar nature of the RPE cells in *M. niger*. Figure 1a represents a radial section of the retina; in such a section most of the long axes of the RPE cells and rod outer segments are visible and they therefore appear elongated. In such sections the additional ‘layer’ between the RPE and rods is not apparent. However, in a more oblique section (Figs 1b and 2a) RPE cells and rod outer segments are not cut parallel to their long axis. The RPE cells therefore appear as several discrete layers and the rod outer segments have a circular profile. In such sections the ‘additional layer’ between the RPE and the rods observed by Brauer^20^ is visible. However, rather than being a layer of specialised structures, it simply represents an oblique section through the inner part of the columnar RPE cells filled with astaxanthin-containing tapetal spheres. This also explains why the structures in this ‘layer’ have no nuclei, as these are located sclerally within RPE cells.

To investigate the composition of the outer retina further we performed TEM EELS imaging in order to study the relative amounts and distribution of sulphur, phosphorus and calcium. The structures we identified as tapetal spheres on the basis of light and electron microscopy (Figs 1 and 2) did not contain any of these elements (see Supplementary Fig. S2), suggesting low protein and/or nucleic acid content in these structures. In contrast, melanosomes contained above background levels of both phosphorus and sulphur.

SYTOX green stains nucleic acids in cells with permeable membranes. In the *Malacosteus* retina, as expected, it stains the nuclear layers of the neural retina (see Supplementary Fig. S3). Staining of RPE nuclei is masked by the melanin within the RPE. There is no indication of staining elsewhere in the retina.

### Absorption spectrophotometry of cryosections

The spectral absorption of small circular areas within 8 different layers of the *Malacosteus* retina, from the RPE to the inner retina, was determined in 5 different retinal cryosections. Every one of the 5 transverse spectral transects showed a similar pattern of absorbance and all scans for a given retinal layer were therefore averaged (Fig. 3). All layers measured showed an absorbance peak close to 673 nm with other maxima at shorter wavelengths. Although it is not possible to be certain of the exact nature of the pigments causing these absorbance spectra, the multiple absorbance peaks are characteristic of chlorophyll-derived substances. Comparison of the scans obtained here with those of purified bacteriochlorophyll extracted from the *Malacosteus* retina previously (see Supplementary Fig. S4) show the absorption spectra to be very similar.

**Figure 4:**
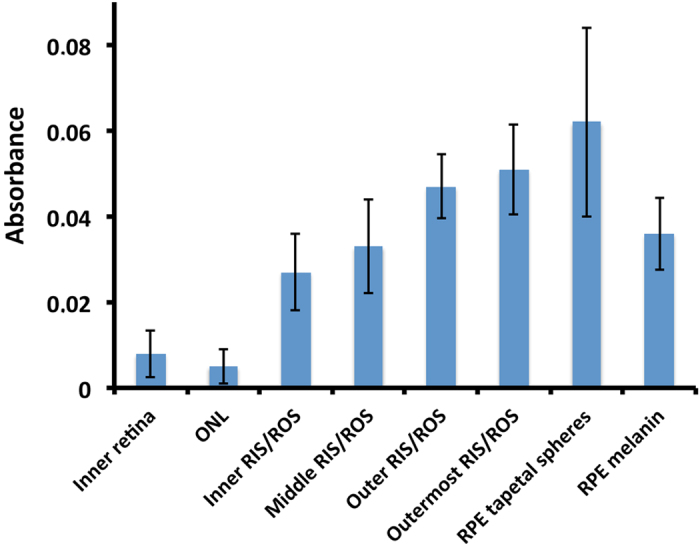
Density of the chlorophyll-like substance in different layers of the *Malacosteus niger* retina determined by absorption spectroscopy of retinal cryosections. The absorbance at 673 nm due to the chlorophyll-like substance in different retinal layers, was estimated by taking the absorbance at this wavelength and subtracting the background absorbance (calculated by taking the average of the absorbance 25 nm either side of 673 nm). In this way the effect of absorbance not due to the chlorophyll-like substance, such as astaxanthin, was greatly reduced. The data shown for the eight transect positions, are averages (+/−SD) from cryosections taken at five different retinal locations. The transect positions are shown in Fig. 3. The highest concentration of the bacteriochlorophyll is at the RPE/ROS border in the region containing the tapetal spheres, although one cannot assume it is actually in the spheres rather than the ROS. All labels as in Fig. 1.

Rather surprisingly, the chlorophyll-like substance was found throughout the *M. niger* retina. Judged by the height of the longwave (673 nm) absorption maximum (measured relative to local background absorption) its density was lowest in the inner retina and outer nuclear layer (ONL) and steadily increased as the outer retina was traversed, reaching a maximum at the RPE/ROS border (Figs 3 and 4). As scans approach the RPE, the absorbance of the red tapetum was clearly apparent as a shortwave-absorbing cut-off filter superimposed on the chrorophyll-like absorption spectrum (Fig. 3).

### Scanning laser confocal fluorescent microscopy

**Figure 5:**
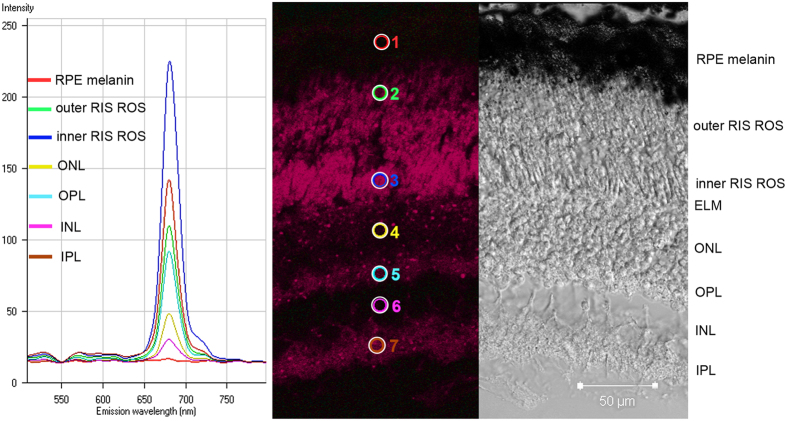
Fluorescence emission spectra from different regions of a *Malacosteus niger* retina. The left panel shows the fluorescence spectra recorded from the different regions of a retinal section. The middle panel shows a fluorescence image resulting from excitation at 488 nm, with locations (circles) from which emission spectra were recorded. The right panel shows a DIC image of the same field of view. The emission spectra have characteristics typical of chlorophyll-like compounds. Fluorescence is most apparent in the rod outer and inner segment layers and the inner and outer plexiform layers. The signal is weaker from other layers of the retina. All labels as in Fig. 1.

**Figure 6:**
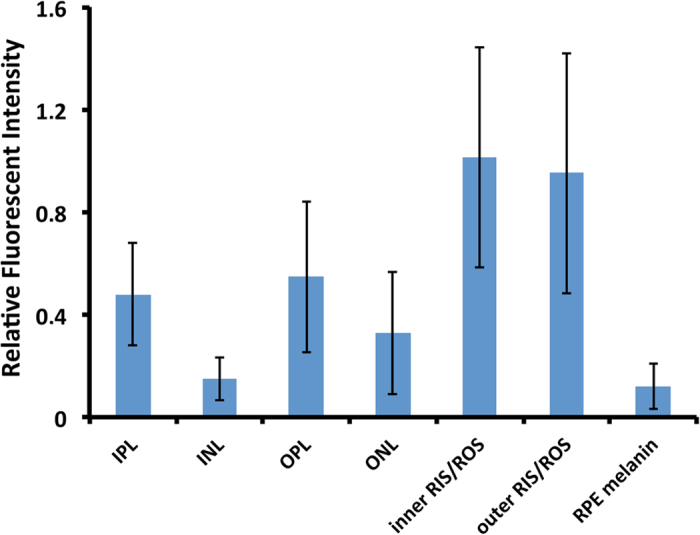
Relative fluorescent intensity of retinal layers of *Malacosteus niger.* Retinal fluorescence (above baseline levels) in different retinal layers averaged for five transects. Fluorescent intensity for each transect was made relative to the average fluorescence of the inner and outer segments for that transect. Means are shown +/−s.d. Fluorescence, indicative of the concentration of putative bacteriochlorophyll c&d, is highest in the RIS/ROS layers, OPL and IPL, and lower in the RPE, INL and ONL. All labels as in Fig. 1.

Fluorescence emission spectra following excitation at 488 nm were recorded for 7 different retinal layers in 5 different retinal cryosections. All emission spectral transects obtained from the 5 sections were similar and only one is shown (Fig. 5, left panel). There was little, if any, fluorescence attributable to aldehyde fixation, which would appear at shorter wavelengths than the observed emissions. Instead, all layers show emission spectra characteristic of a chlorophyll-like substance^15^ with an emission peak at ca. 680 nm (Fig. 5, left panel), seen as red light (Fig. 5, middle panel). The fluorescence intensity varied between retinal layers (Fig. 6), being highest in the inner and outer segments and the inner and outer plexiform layers (IPL and OPL), and lower in other layers. This observation is largely correlated with the distribution of bacteriochlorophyll noted above based on absorption spectroscopy (Figs 3 and 4). The major difference is that the melanin in the RPE masks the fluorescence of any chlorophyll-like substance in this layer. Use of the confocal laser scanning microscope, however, allows greater resolution of its distribution within the inner retina. The chlorophyll-like substance is clearly primarily located in the photoreceptor inner and outer segments and the plexiform layers and is largely absent from nuclear regions. Nevertheless, varying amounts of putative bacteriochlorophyll are found throughout the retina (Fig. 6).

In order to ensure this fluorescence was specific to *Malacosteus*, the retina of another deep-sea species, *Scopelarchus analis*, was also examined. It showed no sign of chlorophyll-like longwave fluorescent emissions (see Supplementary Fig. S5).

### Genetic analysis

A transcriptome of the *M. niger* retina was analysed to identify upregulated genes in comparison to the related *Pachystomias microdon*, in particular those of prokaryotic origin, or associated with bacteriochlorophyll or light sensitivity.

#### Differential gene expression analysis

Following the filtering out of reads of rRNA origin, quality trimming and adapter removal: 2,823,497 paired-end reads remained for the 1^st^ *Malacosteus* sample (MN1), 10,480,590 for the 2^nd^ sample of this species (MN2) and 4,737,894 for the single *Pachystomias* sample (PM1). Following *de-novo* assembly the reference retinal transcriptome comprised 134,989 ‘genes’ (N50 = 866), with 165,493 dependent putative isoforms (N50 = 613). Bowtie alignment of the three samples to the retinal transcriptome resulted in success rates of 70.81% for MN1 (1,999,245 reads with one reported alignment), 75.49% for MN2 (7,911,829 reads aligned), and 33.88% for PM1 (1,605,068 reads aligned). 182 ‘genes’ were found to be significantly up-regulated in *Malacosteus* relative to *Pachystomias* (see Supplementary Table S1). BLASTX homology searches of these gene sequences revealed 122 to be of likely metazoan origin, 56 had no match in either swissprot or Uniref90 databases, and only 4 genes were likely to be of non-metazoan origin. Of the putative non-metazoan genes, two were of likely viral origin (DN71259_c1_g1, DN70242_c1_g2), one of likely fungal origin (DN67159_c0_g1) and one of bacterial origin (DN67491_c7_g1). Gene DN67491_c7_g1 had greatest similarity with the sequence for Polysialic acid O-acetyltransferase from *Escherichia* sp. and showed log^2^ fold changes in expression of 12.29 in *Malacosteus* relative to *Pachystomias* (FDR = 0.021). Whilst other taxonomic groups within the Gammaproteobacteria are known to produce bacteriochlorophyll, species within the Enterobacteriaceae are not known to be photosynthetic. At the isoform level, 236 transcripts were significantly up-regulated in *Malacosteus*: 169 of these most closely matched metazoan sequences, 64 showed no close match and 3 transcripts were of likely non-metazoan origin (see Supplementary Table S2). Of the non-metazoan taxa, one isoform was of likely bacterial origin: DN67159_c0_g1_i2. The closest sequence match to this isoform was L-threonine ammonia-lyase characterized from *Geobacter* sp. from the Deltaproteobacteria: a bacterial group not known to be photosynthetic. This isoform showed log^2^ fold changes in expression of 11.91 in *Malacosteus* relative to *Pachystomias* (FDR = 0.027), however expression of this isoform was only detected in sample MN2. Overall, as shown in Supplementary Table S3, the top twelve gene ontology (GO) terms showing the most significant enrichment in the *Malacosteus* up-regulated gene and isoforms sets were all representative of biological processes involved in the response to, and perception of stimuli, with specific reference to light (i.e. GO:0007601 visual perception; GO:0050953 sensory perception of light stimulus; GO:0007600 sensory perception; GO:0009583 detection of light stimulus; GO:0050877 neurological system process; GO:0009416 response to light stimulus; GO:0007602 phototransduction; GO:0009581 detection of external stimulus; GO:0009582 detection of abiotic stimulus; GO:0009584 detection of visible light; GO:0051606 detection of stimulus; GO:0001750 photoreceptor outer segment–only gene associated GO terms presented).

#### Metatranscriptomic Taxonomic Assignment

*De-novo* assembly of the metatranscriptomic read sets resulted in 135,834 ‘genes’ (N50 = 875), with 167,107 dependent putative isoforms (N50 = 610) for the combined *Malacosteus* samples transcriptome. For the *Pachystomias* transcriptome, 49,363 ‘genes’ (N50 = 511), with 52,947 dependent putative isoforms (N50 = 465) were assembled. In MEGAN, 66,998 and 30,212 transcripts at the isoform level could be assigned to a specific taxonomic clade for the *Malacosteus* and *Pachystomias* read sets respectively. Following square root normalization this resulted in 3,098 and 2,209 assigned reads for each retina metatranscriptome. Overall, systematic profiles comprising the taxonomically assigned transcripts from the retinae of *Malacosteus* and *Pachystomias* were broadly similar (see Supplementary Table S4). Using the current version of the NCBI taxonomy, focusing on level 4 in the taxonomic hierarchy, the taxon with the highest levels of assigned transcripts in both *Malacosteus* and *Pachystomias* were the Metazoans (75.53% & 69.58% respectively). This was followed by the Gammaproteobacteria (4.77% & 5.93%), Bacili (3.58% & 5.3%), Actinobacteria (4.61% & 2.32%), Fungi (2.3% & 1.58%) and Streptophyta (2.3% & 1.58%). Directed homogeneity tests found no significant differences between the *Malacosteus* and *Pachystomias* retinas for known photosynthetic bacterial classes: Gammaproteobacteria (148 *vs*. 131 assigned reads *P* = 0.68), Alphaproteobacteria (42 *vs*. 45 *P* = 0.88), Chloroflexi (not recovered), Chlorobi (1 *vs*. 0* P* = n/a), Clostridia (*Heliobacteria*)(41 vs. 30 *P* = 0.22) and Acidobacteria (*Chloracidobacterium*)(0 *vs*. 2 *P* = n/a).

#### GO-term enrichment analysis of transcripts of putatively bacterial origin

Assembly of reads of putative bacterial origin that aligned successfully against sequences in the RefSeq bacterial genome database resulted in 117 and 128 assembled transcripts from the *Malacosteus* and *Pachystomias* retinae respectively (N50 = 378 & 330). BLASTx homology searches for the RefSeq datasets were successful for 114 of the *Malacosteus* transcripts and 111 of the *Pachytomias* transcripts. Of these, GO annotation was recovered for 39 of the *Malacosteus* and 30 of the *Pachystomias* transcripts. For the reads successfully aligned against the BC custom database, Trinity assembly resulted in 18 and 32 transcripts from the *Malacosteus* and *Pachystomias* retinas respectively (N50 = 1201 & 453). BLASTx homology searches were successful for 10 of the *Malacosteus* assembled transcripts with additional GO annotation for 5 of these. For the *Pachystomias* BC dataset, BLASTx searches found hits for 31 transcripts, a further 15 having GO annotation. Functional enrichment analysis found no significant over-representation of any GO terms in the annotation of *Malacosteus* transcripts in comparison to those from *Pachystomias* for either the RefSeq or BC database aligned read sets. GO searches for ‘bacteriochlorophyll’ identified 11 directly associated terms. An additional 51 other GO terms that have been co-annotated with these terms in at least one previous study were also recovered (see Supplementary Table S5). Visual inspection of the annotation for the four custom database transcript sets revealed none of the GO terms directly associated with bacteriochlorophyll and only overlapped with eight of the other co-annotated terms. All these overlapping GO terms were for general cellular components, molecular functions or biological processes and showed very low levels of specific association with any of the GO terms directly related to bacteriochlorophyll (probability similarity ratio (S%) = 0.01–0.05) (Supplementary Table S5).

All raw transcriptomic reads used in this study have been archived in the NCBI Sequence Read Archive (SRA): accession SRP094458.

## Discussion

Bacteriochlorophylls c&d are only known to be produced by photosynthetic green sulphur bacteria. The way that these compounds become accessible to *Malacosteus*, ultimately to be sequestrated as a retinal photosensitizer, may involve ingestion from the stomiids’ food. This possibility is supported by measurements of the fluorescence spectra of the retinal photosensitizer and the gut contents of *M. niger*, both of which showed characteristics of bacteriochlorophyll and were similar to spectra derived from the copepods on which *M. niger* feeds^17^.

However, a possible alternative source suggested itself to us based on some unusual structures between the RPE and the rods of *Malacosteus indicus* observed by Brauer^20^ (see Supplementary File S1). We wondered if these unusual structures, seemingly unique to *Malacosteus*, might be the site for endosymbiotic bacteria. Indeed, these structures appear as a discrete layer of anucleate ‘cells’ between the RPE and the rod outer segments in some of our sections (Figs 1b and 2) and seem ideally placed to provide bacteriochlorophyll to the photoreceptor outer segments. Furthermore, they contain spheres that are approximately the correct size, shape and ultrastructural appearance to be bacteria (Fig. 2). However, the data presented here do not support such a conclusion.

If the novel structures described by Brauer^20^ truly were the site of an accumulation of endosymbiotic bacteria, the bacteriocholophyll should be concentrated here and the outer segments. Although, bacteriochlorophyll is indeed abundant in this region of the retina (Figs 3, 4, 5, 6), it is found in other layers of the retina too. In fact, the unusual structures described by Brauer^20^ are most simply explained as an artefact of oblique sectioning of unusually columnar RPE cells, rather than representing a specialised layer of novel structures located between the RPE and ROS. Based on light and electron microscopy, RPE cells of *M. niger* contain only two inclusions; melanosomes and astaxanthin-containing tapetal spheres. The tapetal spheres do not contain high levels of S, P or Ca (see Supplementary Fig. S2), which is compatible with the notion that they predominantly consist of high concentrations of astaxanthin (C_40_H_52_O_4_), which lacks these assayed elements. In contrast melanosomes are made up of melanin, proteins and phospholipids which would explain the presence of S, and P^24^. Further evidence that there are no bacteria in the outer retina of *M. niger* is provided by the SYTOXgreen staining, which only located nucleic acids in the nuclear regions of the retina (see Supplementary Fig. S3). There was no indication of staining elsewhere that might suggest the presence of prokaryotic nucleic acid.

However, the most convincing evidence for the lack of endosymbiotic bacteria within the *M. niger* retina comes from the analysis of mRNA transcripts. Although these reveal ample evidence of bacterial RNA in the retina of *M. niger*, the same was true for *P. microdon*, whose retina does not contain the photosensitizer. This suggests that these bacteria represent contamination and are not specific to *M. niger*. The only genes uniquely upregulated in *M. niger* are not connected with bacteria (see below). The molecular analysis also provided no evidence for the presence of genes involved in the synthesis of bacteriochlorophyll specific to the *M. niger* retina.

We therefore conclude that the initial suggestion that the photosensitizer in the retina of *M. niger* has a dietary origin^17^, is probably correct and that there is no evidence for endosymbiotic bacteria in the eye of this species that might provide the bacteriochlorophyll.

To act as a photosensitizer the photosensitizing pigment must obviously be located in the photoreceptor outer segment in very close proximity to the visual pigment. Its localisation in the outer segment by microspectrophotometry of isolated cells^9^,^10^ and here in cryosection, shown by both absorbance spectrophotometry (Figs 3 and 4) and laser scanning confocal fluorescence microscopy (Figs 5 and 6), is therefore no surprise.

However, what is surprising is to find it throughout all retinal layers in varying amounts. It is difficult to believe the bacteriochlorophyll has any physiological function in the inner retina and its presence in the retinal plexiform layers might simply be a consequence of the fact that chlorophyll-like molecules are very lipid-soluble and the plexiform layers (compared to the nuclear layers) contain a higher density of lipid-based membrane.

The high density of the chlorophyll-like pigment in the areas scleral to the ROS (Figs 3 and 4) could have a number of explanations. Firstly, the RPE could act as a storage region for bacteriochlorophyll, just as it does for the visual pigment chromophore. Alternatively, as the astaxanthin of the tapetal spheres is contained in lipid droplets^9^, as in the cone oil droplets of birds and reptiles^25^,^26^, and as chlorophyll is very lipid soluble it may preferentially accumulate here. Unfortunately, the spatial resolution of our spectroscopy and the masking of the bacteriochlorophyll absorbance spectra by astaxanthin does not allow us to conclusively locate it to individual tapetal spheres, but only to the retinal region where they are located.

With respect to function, there is little doubt that bacteriochlorophylls have the ability to enhance longwave sensitivity in *Malacosteus*^14^,^15^. Perhaps more surprisingly, various chlorophyll-related compounds, such as chlorine e6, will interact with bovine rhodopsin to act as a photosensitizer for long wavelength illumination^27^, as well as enhancing thermal stability of the visual pigment^28^. Furthermore, they are also accumulated by isolated salamander rods^29^ and in the mammalian retina following intravenous injection^30^,^31^, where they increase sensitivity to longwave illumination. These observations suggest that chlorophyll derivatives become easily incorporated into the retina of many vertebrates and interact with their visual pigments, even those species that do not usually contain such substances.

The pool of transcripts sequenced in this study are representative of all the genes actively expressed at a given time point in the retinas of *M. niger* and *P. microdon* and thus reflect ongoing key cellular processes at the time of tissue collection. Interpretation of patterns of interspecific differential expression identified in this study have to be treated with caution due to potential biases arising from the very low levels (absence in the case of *P. microdon*) of individual replication and potential sequence divergence of *P. microdon* transcripts from homologous loci in the *M. niger* reference transcriptome, both of which could result in false positives being included in the set of genes/isoforms identified as having significantly different levels of expression. Nevertheless, highly significant enrichment in GO terms associated with light stimulus response and the phototransduction pathways (see Supplementary Table S3 for details) were found in the suite of genes/isotopes identified as significantly up-regulated in *M. niger* relative to *P. microdon*. We can therefore be confident that these patterns represent broad overall differences in gene expression for genes associated with such gene ontologies between the retinas of the sampled animals.

What is causing these systematic differences in interspecific gene expression? One hypothesis is that patterns of retinal gene expression are strongly influenced by the visual ecology of the two species. Despite both deep sea fish being members of the same small clade of derived stomiids^32^, *M. niger* and *P. microdon* differ in their trophic ecology and habitat preference: *P. microdon* undertaking diel vertical migrations (DVM) over a large depth range in search of its myctophid (lanternfish) prey, similar to most other members of the clade; *Malacosteus* species, generally remaining at depth (>600 m) and feeding largely on copepods^33^,^34^. However, it remains unclear how such interspecific differences in visual ecology could drive the differential expression of genes in the phototransduction pathway in *M. niger* relative to *P. microdon*.

An alternate hypothesis is that patterns of differential expression are instead more strongly influenced by the intracellular environment and the presence of retinal bacteriochlorophyll in *M.niger* The upregulated genes might be involved either in the unique bacteriochlorophyll-associated transduction pathway that appears to operate in this genus^1^,^14^,^15^,^17^, or to the highly reactive nature of the bacteriochlorophyll photosensitizer^35^. The latter hypothesis would suggest that differences in retinal gene expression patterns for genes related to light reception and cell signalling may be driven largely by the need to replace cellular components with these functions that are damaged by the oxidising action of bacteriochlorophyll. Support for this hypothesis may come from the high quantities of astaxanthin, a xanthophyll keto-carotenoid^8^,^9^,^36^ (most likely of dietary origin), found in the *Malacosteus* retina.

Chlorophylls in their excited state may be effective photosensitizers, but they can also react with molecular oxygen to produce singlet oxygen, which is a powerful oxidising agent capable of damaging or killing cells exposed to it. Carotenoids are able to prevent the harmful effects of singlet oxygen by either quenching it or preventing its production^35^,^37^. Thus, carotenoids often play a role as photoprotective agents and photosynthetic plants and bacteria frequently contain them in close association with their chlorophylls. In addition, the antioxidant properties of polar carotenoids derived from marine bacteria, including astaxanthin, are protective of phosphatidylcholine membranes^38^. A similar function has been ascribed to the primate macular pigment in the (non-chlorophyll-based) visual process^39^.

It is therefore possible that the unusual, astaxanthin-based, tapetum of *M. niger* serves not only to enhance sensitivity to long wavelengths, but also negates the potentially harmful effects involved in photosensitization by bateriochlorophyll derivatives.

## Materials & Methods

### Animals & tissue preservation at sea

Light and electron microscopy was performed on two specimens of *M. niger*; one captured during *FS Sonne* cruise 142 to the Musician Seamounts on the Hawaiian ridge of the North Pacific and one during *RRS Discovery* cruise 243 to the Eastern North Atlantic. Tissue was fixed in 2% paraformaldehyde in 0.13 m phosphate buffer pH 7.4 for 2–4 h before being rinsed in buffer for a further 2–4 h. It was then transferred to 30% sucrose in buffer until the tissue sank (12–24 h) after which the material was transferred to fresh 30% sucrose and frozen for transport.

Another specimen of *M. niger*, used for cryosection spectroscopy and SYTOXgreen staining was obtained during cruise ANT-XXIV/1 of the *FS Polarstern* in the Eastern North Atlantic. Immediately after capture enucleated eyes were stored at −80 °C and transported to our laboratory on ice. Two further *M. niger* specimens, and a single *P. microdon,* were caught using the NOAA ship *Pisces* during cruise 14–04 to the Bear and Physalia Seamounts in the Western North Atlantic. Immediately after capture, retinae were rinsed in RNAlater and stored in fresh RNAlater at −20 °C and transported to our laboratory on dry ice, to be used in transcriptome analysis.

A non stomiid deepsea fish, *Scopelarchus analis*, also used for cryosection spectroscopy, was caught on *FS Sonne* cruise 142 and preserved and processed as the other specimens from this cruise (see above).

All animals were dead on capture and all subsequent procedures involved preserved or frozen tissue.

### Light & electron microscopy

After thawing, six pieces of central and peripheral retina were excised and fixed in 4% paraformaldehyde and 2.5% glutaraldehyde in 0.1 m phosphate buffer with pH 7.4. After thorough rinsing in buffer three blocks were postfixed in osmium tetroxide (2%) in the same buffer for 2.5 h. The blocks were rinsed again, dehydrated in a graded series of alcohol and embedded in Epon.

For light microscopy 1–2 μm sections of non-osmicated tissue were stained according to Richardson^40^ and photographed with a digital camera mounted on an Axioscope (Carl Zeiss Microscopy GmBH, Jena, Germany). For standard electron microscopy 60 nm sections of osmicated tissue were prepared, the contrast of most sections was enhanced with lead citrate and viewed with a LEO 912 electron microscope (Carl Zeiss NTS, Jena, Germany).

The electron microscope was equipped with an omega filter which allowed sections to be irradiated with near monochromatic electron beams. Using electron energy loss spectroscopy (TEM EELS; acceleration voltage 80 kV) allowed the visualisation of the amount and localisation of the elements phosphorus, sulphur and calcium in tissue sections, indicative of proteins and/or nucleic acids. For this analysis 30 nm sections of non-osmicated tissue were used and image processing and subtraction performed with analySIS 3.0 software (Soft Imaging Systems GmbH, Münster, Germany).

### Cryosectioning

Since cryosectioning of the native tissue proved impossible, the frozen eyes were carefully thawed in 4% paraformaldehyde (in PBS) for 4 h. The eyeballs were opened to remove the lens and cornea, transferred to 30% sucrose and mounted with TissueTec for cryosectioning at 10, 20, and 30 μm using an HM560 cryostat (Microm GmBH, Jena, Germany). Some sections were stained with SYTOX green for DNA visualisation (dilution in PBS at 1:50,000 for 20–30 min). The remainder were used for spectrophotometry.

### Absorption spectroscopy

The spectral absorption (178–880 nm) of small (ca. 15 μm diameter) circular areas of the retinal cryosections was determined using a 600 μm fibre optic linked to an S2000 CCD spectrometer (Ocean Optics Inc., Dunedin, Florida) using the basic method outlined in Hantz *et al*.^41^. Sections were viewed using a x40 objective on a Zeiss Axioskop microscope with the end of the fibre optic positioned in the focal plane of the attached camera mount. To localise the area being sampled by the fibre optic, light was shone down it while looking through the eye pieces of the microscope to view the projected light spot. To measure the spectral absorption of the illuminated area, the light source was removed from the end of the fibre optic, which was then coupled to the S2000 spectrometer, itself connected to a notebook PC, running Ocean Optics OOIBase32 software with which spectra were recorded as raw CCD counts. In this configuration, following a reference scan of an area of the slide without tissue, the spectrometer was used to measure the spectral modification of the microscope’s internal light source by the chosen area of the cryosection. Spectral absorbance was calculated using MS Excel from data recorded at points on transects of the retina at five different locations. For each transect, the spectral absorption was determined for eight distinct layers of the retina: (1) an area of the RPE near Bruch’s membrane containing mainly melanin, (2) the inner edge of the RPE dominated by red tapetal pigmentation, (3) the distal margin of ROS layer ca. 15 μm from the tapetum, (4) an area of the outer ROS layer ca. 30 μm from the tapetum, (5) the middle of the ROS layer, (6) the base of the ROS layer near the ELM, (7) the ONL, and (8) the inner retina (the resolution of the cryosection was not sufficient to allow identification of the precise inner retinal layer being sampled). 5 different cryosections were examined in this way.

### Scanning laser confocal fluorescence microscopy

10 μm unstained cryosections were first viewed in a LSM 510 META confocal microscope (Carl Zeiss Microscopy GmBH, Jena, Germany) with differential interference contrast (DIC) illumination. A λ-stack analysis was performed on 5 different sections excited at 488 nm and fluorescence recorded in ca.10 nm bins. Fluorescence emission spectra were recorded for the: (1) RPE, (2) outer ROS layer, (3) inner ROS layer, (4) ONL, (5) OPL, (6) INL, and (7) IPL.

### Genetic analysis/molecular biology

Retinae were dissected from the RNAlater-stored eyes of each of the three sampled stomiids (two *M. niger* (MN1, MN2) and a single *P. microdon* (PM1)). Retina tissue-specific total RNA was extracted from each individual separately, using the RNeasy^®^ Mini Kit (Qiagen, Valencia, California) standard protocol, including DNase purification. Quantity and quality of extracted RNA was assayed for each sample using an Agilent 2100 Bioanalyzer with RNA6000 Nano chip kit (Agilent Technologies, Inc., Santa Clara, California). Bar-coded, strand-specific cDNA libraries were prepared for each individual using the TruSeq^®^ Stranded RNA sample preparation kit (Illumina, San Diego, California), according to manufacturer’s instructions. Ribo-Zero Gold rRNA depletion was carried out prior to cDNA synthesis. Individual cDNA libraries were diluted to 2 nM, and 5 μl from each library was pooled for multiplexing. 6 pM of this pooled sample was added to each of three flow cell lanes on an Illumina HiSeq 2500 sequencer, generating 100 bp paired-end reads. Read quality trimming and sequencing adapter removal were carried out using Trimmomatic v3.3^42^. Using a sliding window of 5 bp, reads were trimmed where the average Phred quality score dropped below 30. Reads below a minimum length of 50 bp were removed.

#### Differential gene expression analysis

Prior to quality trimming and adapter removal, any remaining reads of rRNA origin were filtered out using SortMeRNA V2^43^. As no close reference transcriptome/genome exists for stomiid fish, reads were first *de-novo* assembled into a new reference transcriptome using Trinity V2.1.1^44^. Reads from only the *Malacosteus* individuals (MN1, MN2) were used for transcriptome assembly.

Functional annotation of the reference transcriptome was carried out using the Trinotate v2.0.2 workflow (http://trinotate.github.io/). Briefly, TransDecoder v2.0.1 (http://transdecoder.github.io/) was used to identify candidate coding regions within the assembled contigs. Separate BLASTx and BLASTp searches of the constituent contigs making up the reference transcriptome against the SwissProt and Uniref90 databases were then carried out (cut off e-value: 1e-5). Protein domains, transmembrane spanning domains and signal peptides were identified using HMMER-2.3.2 (http://hmmer.wustl.edu/), tmHMM-2.0c^45^ and SignalP 4.0^46^ respectively.

To estimate transcript abundance, reads from each of the three samples (MN1, MN2, PM1) were first aligned separately against the *Malacosteus* reference transcriptome using Bowtie 1.1.2^47^. Abundance estimates at the gene and isoform level were then calculated in RSEM v.1.2.25^48^. Expression values were normalized across samples using Trimmed Mean of M-values (TMM), followed by differential expression analysis, both in edgeR 3.12.0^49^. For differential expression analysis, the samples MN1 and MN2 were treated as biological replicates within the sample type *Malacosteus*. As only one sample of *Pachystomias* was available (PM1), the dispersion parameter could not be estimated from the read data and was set to 0.4. As this differential expression analysis was explorative, a relatively low FDR threshold of 0.05 was used to identify genes significantly up-regulated in the *Malacosteus* samples relative to *Pachystomias*. Gene ontology (GO) categories significantly enriched in the *Malacosteus* up-regulated gene set were also reported.

#### Metatranscriptomic Taxonomic Assignment

For Metatranscriptomic analyses, reads obtained from both *Malacosteus* retina samples were again used to *de-novo* assemble a reference transcriptome in Trinity. A separate *Pachystomias* retina reference transcriptome was also assembled. For both transcriptomes, putative rRNA reads were retained to increase the potential pool of non-stomiid reads available for species assemblage analyses. BLASTx homology searches of the NCBI-NR database (downloaded 15.10.15) were carried out for the assembled contigs of each reference transcriptome, with an E-value cut off of 1e-04. The results of the BLASTx surveys were fed into MEGAN V5.10.6^50^ for comparative taxonomic analysis. To compare the assigned taxonomic content of the assembled contigs from the *Malacosteus* and *Pachystomias* retinal tissue, the overall number of transcripts analysed for each retina ‘environment’ were square-root normalized. Directed homogeneity tests were then carried out to test if there were significant differences between the two species’ retinae in the number of transcripts assigned to identified bacterial groupings at the taxonomic rank of class.

#### GO-term enrichment analysis of transcripts of putatively bacterial origin

Not all sequenced transcriptomic reads could be assembled/aligned in the previous transcriptomic analyses, potentially resulting in low copy number bacterial symbiont sequences being discarded unanalysed. To ameliorate this problem, retina transcriptomic reads from *Malacosteus* and *Pachystomias* were aligned separately to two custom databases of bacterial nucleotide sequence data using Bowtie 2 V.2.2.6^51^. To create the first database,. fna files from all bacterial genomes listed as “representative genome” or “reference genome” and “latest” under the NCBI RefSeq Bacteria Genomes FTP site were downloaded and merged (4848 sequence files, downloaded 06.01.16; hereafter referred to as the RefSeq database).

To create the second, a NCBI search for nucleotide sequences with the search term “bacteriochlorophyll” was carried out (3259 sequences, downloaded 06.01.16: hereafter referred to as the BC database). Reads that did not align successfully to these databases were discarded, resulting in four distinct datasets of filtered reads of putative bacterial origin: *Malacosteus* RefSeq, *Pachystomias* RefSeq, *Malacosteus* BC, *Pachystomias* BC. Each of these read datasets were then assembled into transcripts using Trinity. The resulting transcripts for each of the four data sets were functionally annotated in Blast2GO 3.2^52^. BLASTx homology searches were carried out with an E-value cut off 1e-03, with the single highest scoring sequence being retained. To test for over-representation of specific GO terms between paired *Malacosteus*/*Pachystomias* datasets, aligned against the same database, enrichment analysis (Fisher’s exact test, one-sided) was carried out in Blast2GO, using the *Pachystomias* annotation in each dataset pair as the reference. Finally, GO terms resulting from the search “bacteriochlorophyll” on the QuickGO website (http://www.ebi.ac.uk/QuickGO), plus all other GO terms recorded as co-occurring with these terms from previous annotation studies, were collated. The presence/absence of these GO terms among the annotation records for the four datasets was then manually surveyed to search for evidence for the transcription of bacterial genes involved in the synthesis pathways of bacteriochlorophyll within the sequenced retinae.

## Additional Information

**How to cite this article**: Douglas, R. H. *et al*. Localisation and origin of the bacteriochlorophyll-derived photosensitizer in the retina of the deep-sea dragon fish *Malacosteus niger. Sci. Rep.* **6**, 39395; doi: 10.1038/srep39395 (2016).

**Publisher's note:** Springer Nature remains neutral with regard to jurisdictional claims in published maps and institutional affiliations.

## Supplementary Material

Supplementary Material
