# Supplementary material for: Localisation and origin of the bacteriochlorophyll-derived photosensitizer in the retina of the deep-sea dragon fish Malacosteus niger
Source: Sci Rep. 2016 Dec 20;6:39395. doi: 10.1038/srep39395 (PMC5171636; doi:10.1038/srep39395)
Supplement: Supplementary Material [file srep39395-s1.pdf]

Supplementary material

Localisation and origin of the bacteriochlorophyll-derived photosensitizer in the retina of the deep-sea dragon fish *Malacosteus niger*

Ronald H Douglas, Martin J Genner, Alan G Hudson, Julian C Partridge and Hans-Joachim Wagner

Supplementary File S1 & Figure S1 Translation of extracts from Brauer (1908) with associated figures
Supplementary Figure S2 Elemental spectroscopy (TEM EELS) imaging of the *Malacosteus niger* RPE
Supplementary Figure S3 SYTOX green staining of the *Malacosteus niger* retina
Supplementary Figure S4 *In vivo* and *in vitro* absorption spectra of chlorophyll-derived retinal photosensitizer in *Malacosteus niger*
Supplementary Figure S5 Fluorescence emission spectra from different regions of the retina of the *Scopelarchus analis* retina
Supplementary Table S1 Retina genes significantly up-regulated in *Malacosteus* relative to *Pachystomias*
Supplementary Table S2 Retina isoforms significantly up-regulated in *Malacosteus* relative to *Pachystomias*
Supplementary Table S3 Gene ontology terms significantly enriched in genes up-regulated in *Malacosteus* relative to *Pachystomias*
Supplementary Table S4 Taxonomic identity and diversity of assigned reads following normalization of transcript number from the *Malacosteus* and *Pachystomias* retina metatranscriptomes
Supplementary Table S5 Identity of transcripts of putatively bacterial origin from *Malacosteus* and *Pachystomias* retinae overlapping in gene ontology (GO) term annotation with GO terms directly associated with the search term ‘Bacteriochlophyll’ (S% = 100) and those recorded as being co-annotated with these search terms in previous studies (S% < 100)

Supplementary File S1. Translation of extracts from Brauer (1908)

Because the original publication is in German and is hard to obtain, but was central to initiating the current study, we include a partial translation here.

Extract from p177, 3 lines from the bottom of the page, to p178 line 19.
"However, the most interesting thing about the Malacosteus *eye is the following. Between the rods and the pigment cells there is a very strange layer (Fig 14-16, ta;* [see Supplementary Fig S1]*). It consists of yellowish structures. They have a quite irregular shape. They are most strongly developed in the region of the optic nerve head (op) where they are sometimes very large. Going sideways from here (i.e. peripherally) they decrease in size. Nasally they decrease in size rapidly and soon disappear completely. In contrast temporally they persist until almost the base of the iris. They are completely homogenous, anucleate, and appear like a solidified secretion. Initially I assumed they were artefacts but had to give up that notion. Firstly it is completely unclear to me where these structures could have come from as the preparation was treated with sublimat* (a fixative)*. If their formation was due to preservation one would have found them in other locations too. Furthermore, their strikingly regular layering speaks against this. In the region of the optic nerve head it was not so obvious, however, further away, where they are not so thick, each structure has the same width as a pigment cell. As one can see especially well under higher magnification by comparing drawings from bleached (Fig 16a) and unbleached (Fig 16b) sections, in some cases the relationship is so intimate that there can be little doubt that the structures are derived from the pigmented cells. The formation of the large irregular structures is possibly due to the fusion or tight agglomeration of smaller structures due to the shrinkage of the eye (during fixation). It must also be pointed out that in the live animal the fundus shines purple. There must therefore be a tapetum in the eye. However, such a structure is missing in the usual location and couldn't function as it would be blocked by the retinal pigment. It is therefore very likely that the aforementioned structures represent the tapetum."*

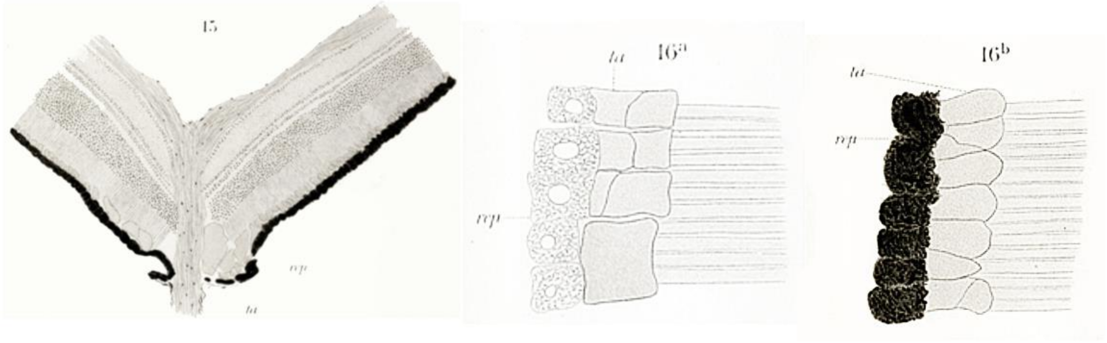

Supplementary Figure S1. plate XXXIII from Brauer (1908)
15 – Eye of *Malacosteus indicus* magnified x135. 16 – Bleached (a) and unbleached sections of the same eye at x810 magnification. rep – retinal pigment epithelium; ta – tapetum. In 16a&b the elongated structures on the right represent rod outer segments.

Brauer, A. (1908) Die Tiefsee-Fische. 2. Anatomische Teil. *Wiss. Ergebn. dt. Tiefsee-Exped. Valdivia* 15, 1-266.

Supplementary Figure S2.

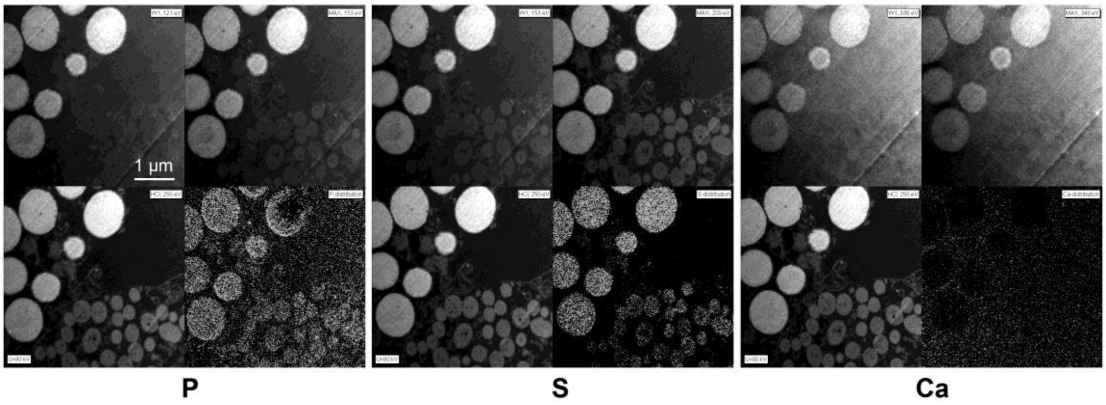

Elemental spectroscopy (TEM EELS) imaging of the *Malacosteus niger* RPE

The large spheres represent melanin granules in the scleral retinal pigment epithelium, while the smaller spheres make up the vitreally located tapetal spheres. The upper left panel of each grid represents the baseline signal, while the upper right panel contains the element-specific emissions signals for phosphorous, sulphur and calcium (P 153eV; S 200eV; Ca 348eV). The lower right panel shows the difference between the previous two images and thus gives the net distribution of the element. The lower left panel is a dark-field image. Melanosomes show clear presence of both P and S, whereas these elements are only slightly above background in the tapetal spheres. No Ca signal was detected in either of the structures.

Supplementary Figure S3.

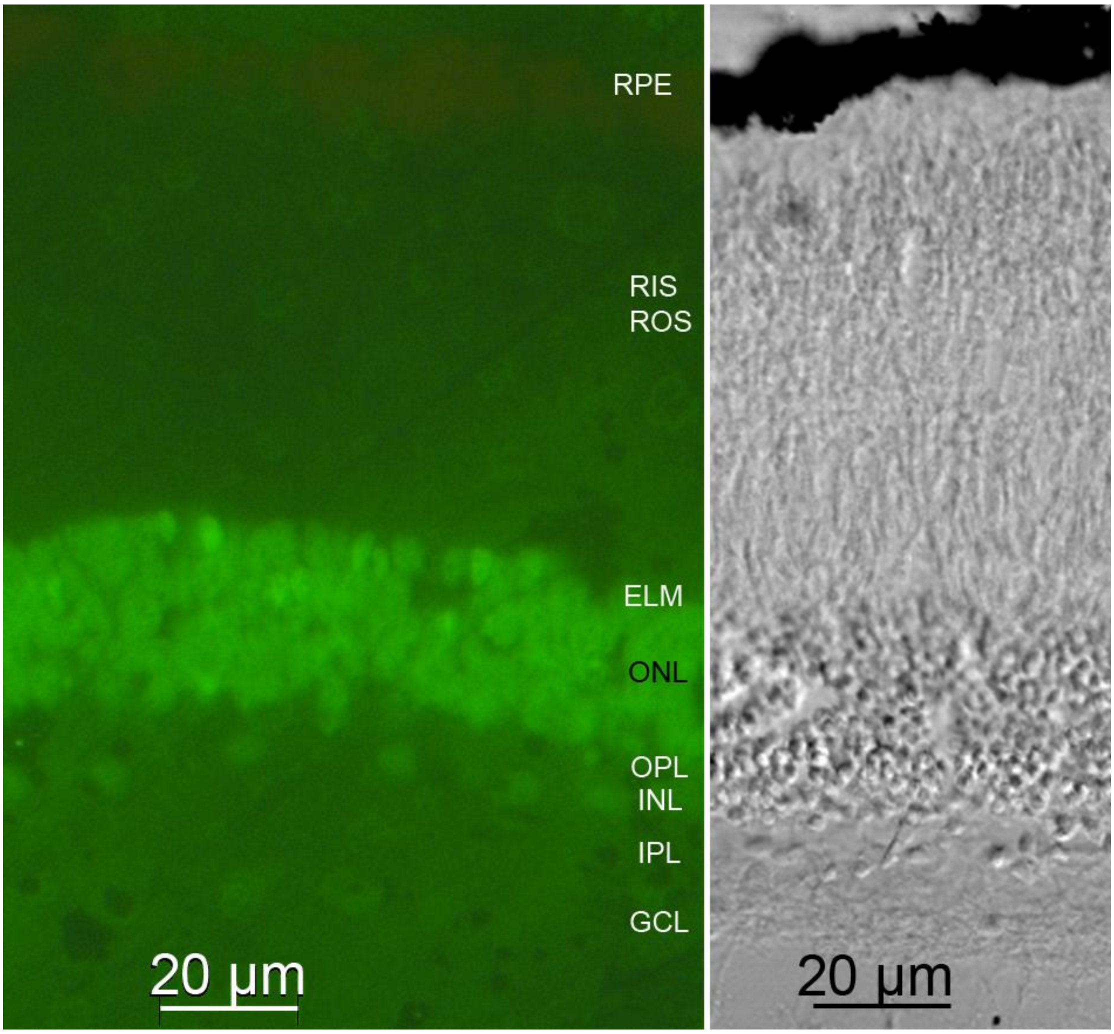

SYTOX green staining of the *Malacosteus niger* retina (left).
Only the nuclear layers (ONL, INL and GCL) are stained, with little fluorescence elsewhere in the retina. The right hand image shows a DIC image of a similar unstained section to show more clearly the retinal layers. Labelling as in Figure 1.

Supplementary Figure S4.

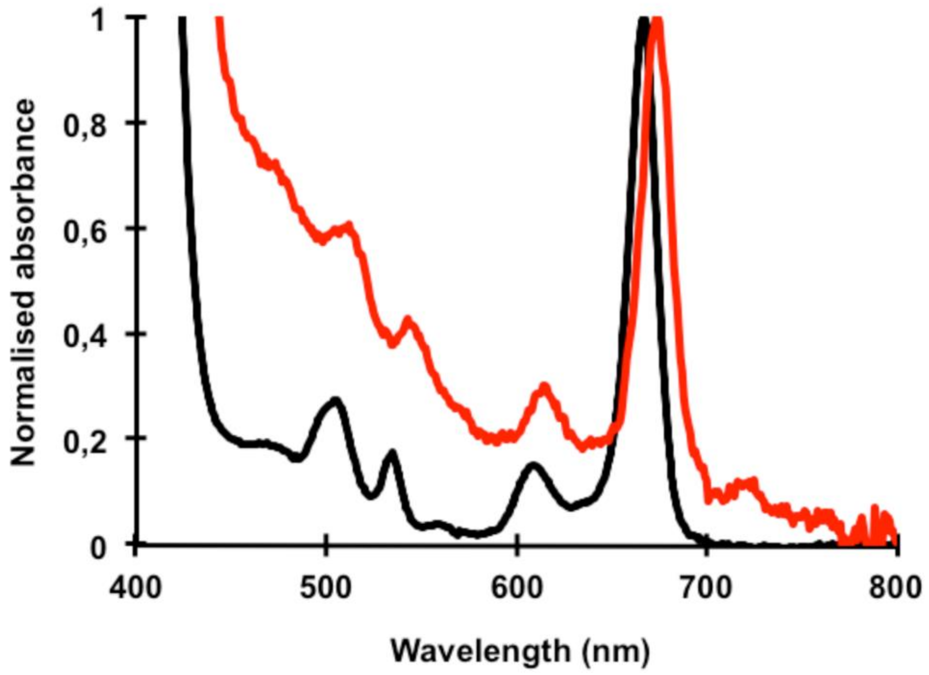

In vivo* and *in vitro* absorption spectra of chlorophyll-derived retinal photosensitizer in *Malacosteus niger

Average absorbance spectrum of the mid and inner RIS/ROS layers from unstained cryosections of *Malacosteus niger* retinae shown in Fig 3 (red) compared to that of the purified diethyl ether extract of a *Malacosteus niger* retinal suspension (black) identified as containing a mixture of defarnesylated and demetallated derivatives of bacteriochlorophylls c and d (Douglas et al., 1999). The two scans, which are very similar, have been normalised to their longwave absorbance peak. The gradual rise in the spectrum recorded here at short wavelengths in the cryosection spectra is due to the encroaching absorption of the red tapetal pigment.

Douglas, R.H., Partridge, J.C., Dulai, K.S., Hunt, D.M., Mullineaux, C.W. & Hynninen, P.H. Enhanced retina longwave sensitivity using a chlorophyll-derived photosensitiser in *Malacosteus niger*, a deep-sea dragon fish with far red bioluminescence. *Vision Res.* **39,** 2817-2832 (1999) .

Supplementary Figure S5.

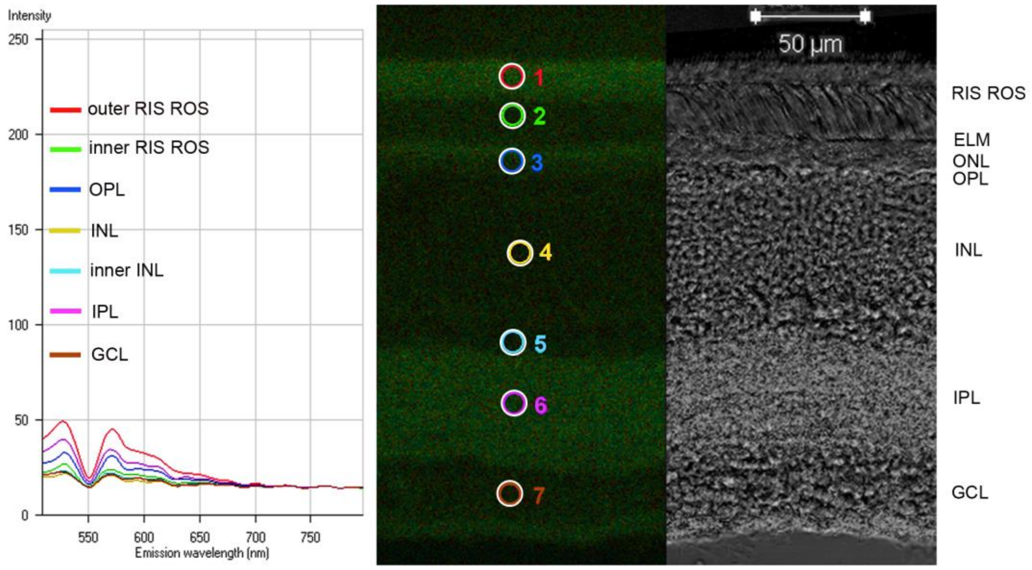

Fluorescence emission spectra from different regions of the *Scopelarchus analis* retina

Fluorescence emission spectra from different regions of a transverse cryosection of the *Scopelarchus analis* retina (left panel), a fluorescence image (middle panel) resulting from excitation at 488 nm, with locations (circles) from which emission spectra were recorded, and a DIC image of the same field of view. This figure is comparable with Figure 5 for *Malacosteus niger*. While the *M. niger* retina showed emission spectra typical of a chlorophyll-like compound, in *S. analis* the only fluorescence is some weak shortwave background autofluorescence and there is no sign of a chlorophyll-like signal.

Supplementary Table S1. Retina genes significantly up-regulated in *Malacosteus* relative to *Pachystomias*. For each gene, annotation with respect to the most similar protein sequence hit from BLASTx searches (Protein Identity) and the organism from which this sequence derived (Taxon) are given. For differential expression analyses, the multi-test corrected significance level (FDR) and normalized expression values per individual (PM1, MN1, MN2) for each gene are also given.

Gene ID	Protein Identity	Taxon	FDR	PM1	MN1	MN2
TRINITY_DN60692_c0_g1	Cytochrome c oxidase subunit 1	Metazoa; Actinopterygii; Salmonidae	0.000110399	0.061	658.729	842.65
TRINITY_DN65229_c0_g1	Guanine nucleotide-binding protein G(t) subunit alpha-1	Metazoa; Mammalia; Muridae	0.000110399	0	579.494	976.686
TRINITY_DN50791_c0_g1	Rhodopsin	Metazoa; Actinopterygii; Stomiidae	0.000113825	1.397	7960.377	8874.18
TRINITY_DN62339_c0_g2	*No Match*	*No Match*	0.000172702	4.375	35655.406	60259.183
TRINITY_DN38768_c0_g3	Retinal rod rhodopsin-sensitive cGMP 3',5'-cyclic phosphodiesterase subunit	Metazoa; Mammalia; Bovidae	0.000192765	0	1210.65	2447.329
TRINITY_DN44906_c0_g1	Guanine nucleotide-binding protein subunit gamma	Metazoa; Actinopterygii; Gasterosteidae	0.000202662	0	1180.021	2069.196
TRINITY_DN34250_c0_g1	NADH-ubiquinone oxidoreductase chain 5	Metazoa; Actinopterygii; Retropinnidae	0.000244866	0	329.225	321.198
TRINITY_DN62973_c1_g2	*No Match*	*No Match*	0.000287407	0	1679.693	1392.302
TRINITY_DN39938_c0_g2	*No Match*	*No Match*	0.000481751	2.843	13909.843	4909.795
TRINITY_DN48042_c0_g1	Cytochrome c oxidase subunit 3	Metazoa; Actinopterygii; Stomiidae	0.000532077	0.196	628.072	692.039
TRINITY_DN69731_c6_g1	Interphotoreceptor matrix proteoglycan 2	Metazoa; Aves; Phasianinae	0.000534892	0	72.615	146.169
TRINITY_DN69457_c2_g1	Riboflavin-binding protein	Metazoa; Actinopterygii; Salmonidae	0.000656198	0	321.865	395.404
TRINITY_DN38768_c0_g2	*No Match*	*No Match*	0.00071397	0	1096.637	2041.104
TRINITY_DN69921_c3_g2	Rod cGMP-specific 3',5'-cyclic phosphodiesterase subunit alpha	Metazoa; Actinopterygii; Ictaluridae	0.000824181	0	202.764	265.114
TRINITY_DN70830_c3_g2	ATP-binding cassette sub-family A member 1	Metazoa; Mammalia; Muridae	0.000916848	0	153.031	125.957
TRINITY_DN45697_c1_g1	Peripherin-2	Metazoa; Aves; Phasianidae	0.001384829	0	270.806	466.878

TRINITY_DN70278_c1_g27	Interphotoreceptor matrix proteoglycan 1	Metazoa; Aves; Phasianidae	0.001488788	0	80.651	92.997
TRINITY_DN57337_c0_g1	Peripherin-2	Metazoa; Mammalia; Canidae	0.001488788	0	169.104	315.598
TRINITY_DN57898_c0_g1	*No Match*	*No Match*	0.002494108	1.973	3041.345	4471.66
TRINITY_DN7106_c0_g1	*No Match*	*No Match*	0.002634897	0	83.276	174.333
TRINITY_DN69026_c5_g1	Retinol dehydrogenase 3	Metazoa; Mammalia; Muridae	0.004317284	0	99.384	132.498
TRINITY_DN54320_c0_g1	Creatine kinase, mitochondrial 2	Metazoa; Actinopterygii; Salmonidae	0.004497442	0	196.397	102.434
TRINITY_DN65214_c0_g1	*No Match*	*No Match*	0.004998362	0	3367.197	91.369
TRINITY_DN64536_c0_g2	*No Match*	*No Match*	0.004998362	0	183.138	359.414
TRINITY_DN59359_c0_g1	Type-4 ice-structuring protein LS-12	Metazoa; Actinopterygii; Cottidae	0.005259391	0	319.357	283.199
TRINITY_DN70660_c2_g1	Prominin-1-A	Metazoa; Actinopterygii; Cyprinidae	0.006326684	0.11	47.325	118.61
TRINITY_DN70565_c0_g2	S-arrestin	Metazoa; Mammalia; Bovidae	0.007089057	0	168.265	50.023
TRINITY_DN53485_c0_g1	NADH-ubiquinone oxidoreductase chain 4	Metazoa; Actinopterygii; Salangichthys	0.007354909	0	118.487	117.57
TRINITY_DN65876_c0_g1	*No Match*	*No Match*	0.007354909	0	115.484	83.751
TRINITY_DN58067_c0_g2	Gamma-crystallin M3	Metazoa; Actinopterygii; Cyprinidae	0.007513501	0	1290.084	2.796
TRINITY_DN70513_c4_g17	*No Match*	*No Match*	0.007513501	0	651.288	688.646
TRINITY_DN64954_c0_g1	*No Match*	*No Match*	0.007513501	0	320.873	325.876
TRINITY_DN71259_c1_g1	Pol polyprotein	Viruses; Retro-transcribing viruses; Retroviridae	0.008163544	0	0.83	39.7
TRINITY_DN63039_c0_g1	Uncharacterized protein	Metazoa; Actinopterygii; Characidae	0.008443126	0	86.811	171.429
TRINITY_DN70717_c0_g1	Si:dkey-52k20.12 protein	Metazoa;Actinopterygii; Cyprinidae	0.0086058	0	37.015	241.88
TRINITY_DN106325_c0_g1	*No Match*	*No Match*	0.0086058	0	2314.773	3.456
TRINITY_DN67162_c3_g1	Voltage-dependent L-type calcium channel subunit alpha-1F	Metazoa; Mammalia; Hominidae	0.0086058	0	20.474	37.709
TRINITY_DN69281_c1_g1	Guanylate cyclase	Metazoa; Actinopterygii; Lepisosteidae	0.0086058	0	58.328	45.807
TRINITY_DN70736_c3_g2	Voltage-dependent calcium channel subunit alpha-2/delta-4	Metazoa; Mammalia; Muridae	0.008687966	0	60.935	81.01
TRINITY_DN67846_c10_g4	Uncharacterized protein	Metazoa; Actinopterygii; Cyprinidae	0.008687966	0	110.325	156.727
TRINITY_DN67613_c0_g1	Uncharacterized protein	Metazoa; Actinopterygii; Gasterosteidae	0.008687966	0	45.187	226.066

TRINITY_DN45697_c1_g2	Peripherin-2	Metazoa; Aves; Phasianinae	0.008687966	0	47.054	148.503
TRINITY_DN70642_c0_g1	Oxygen-regulated protein 1	Metazoa; Mammalia; Cercopithecidae	0.008687966	0	15.739	25.523
TRINITY_DN67838_c0_g2	SEC14-like protein 2	Metazoa; Mammalia; Bovidae	0.008687966	0	36.682	51.461
TRINITY_DN63927_c0_g1	Retinol dehydrogenase 7	Metazoa; Mammalia; Muridae	0.009228132	0	163.539	111.083
TRINITY_DN71136_c1_g2	Calcium-transporting ATPase	Metazoa; Actinopterygii; Gasterosteida	0.00928412	0	15.658	28.074
TRINITY_DN64954_c1_g1	*No Match*	*No Match*	0.00928412	0	343.583	313.246
TRINITY_DN62400_c0_g6	*No Match*	*No Match*	0.009299491	0	1562.288	1.502
TRINITY_DN67660_c0_g1	Signal peptidase complex subunit 3	Metazoa; Actinopterygii; Salmonidae	0.009299491	0	39.595	103.085
TRINITY_DN70849_c3_g1	Retinol-binding protein I, cellular	Metazoa; Actinopterygii; Salmonidae	0.009319606	0	106.609	98.661
TRINITY_DN79162_c0_g1	Synaptotagmin-2	Metazoa; Mammalia; Muridae	0.00987854	0	79.479	117.642
TRINITY_DN70825_c0_g1	Pleckstrin homology domain-containing family A member 5	Metazoa; Mammalia; Hominidae	0.010609874	0	43.302	41.039
TRINITY_DN61263_c0_g1	Guanylyl cyclase-activating protein	Metazoa; Amphibia; Ranidae	0.011037955	0	62.694	58.808
TRINITY_DN3114_c0_g1	Diamine acetyltransferase 1	Metazoa; Actinopterygii; Salmonidae	0.011037955	0	85.395	120.221
TRINITY_DN70896_c0_g1	Photoreceptor-specific nuclear receptor	Metazoa; Mammalia; Bovidae	0.011784287	0	48.714	83.163
TRINITY_DN65277_c0_g1	Homeobox protein otx5-A	Metazoa; Amphibia; Pipidae	0.012075689	0.159	55.189	111.409
TRINITY_DN70958_c5_g1	Protein piccolo;	Metazoa; Mammalia; Hominidae	0.012952422	0	5.808	18.891
TRINITY_DN69281_c0_g1	Retinal guanylyl cyclase 2	Metazoa; Mammalia; Bovidae	0.014367259	0	51.618	30.969
TRINITY_DN58894_c0_g1	Vesicle-associated membrane protein 2	Metazoa; Mammalia; Muridae	0.014367259	0	35.554	36.669
TRINITY_DN69246_c4_g4	Uncharacterized protein	Metazoa; Actinopterygii; Adrianichthyidae	0.016729807	0	1109.174	6.55
TRINITY_DN66896_c0_g1	NADH-ubiquinone oxidoreductase chain 5	Metazoa; Actinopterygii; Retropinnidae	0.016729807	0	14.35	24.708
TRINITY_DN69097_c0_g1	Neuronal pentraxin-1	Metazoa; Mammalia; Muridae	0.01711466	0	20.591	46.141
TRINITY_DN70996_c0_g6	Uncharacterized protein	Metazoa; Actinopterygii; Cichlidae	0.01711466	0	28.149	44.142
TRINITY_DN70519_c4_g1	Protein disulfide-isomerase TMX3	Metazoa; Mammalia; Hominidae	0.017169678	0	51.519	47.643
TRINITY_DN69559_c2_g1	*No Match*	*No Match*	0.017198826	0.27	66.112	97.286
TRINITY_DN53171_c0_g1	Protein lifeguard 2	Metazoa; Mammalia; Hominidae	0.017226679	0	67.708	122.962
TRINITY_DN58241_c0_g1	*No Match*	*No Match*	0.017226679	0	343.277	216.991
TRINITY_DN67290_c0_g4	*No Match*	*No Match*	0.017226679	0	56.515	45.209

TRINITY_DN58487_c0_g1	Ferritin	Metazoa; Actinopterygii; Salmonidae	0.017226679	0	45.304	142.821
TRINITY_DN70398_c0_g6	Cyclic nucleotide-gated cation channel beta-1	Metazoa; Mammalia; Hominidae	0.017226679	0	39.207	38.352
TRINITY_DN68980_c1_g3	Vesicle-fusing ATPase	Metazoa; Actinopterygii; Salmonidae	0.017226679	0	17.84	38.949
TRINITY_DN67159_c0_g1	Threo-3-hydroxyaspartate ammonia-lyase	Fungi; Saccharomycetes; Saccharomycetaceae	0.017226679	0	26.364	39.003
TRINITY_DN58067_c0_g1	Gamma M8b crystallin	Metazoa; Actinopterygii; Nototheniidae;	0.017226679	0	663.852	1.294
TRINITY_DN69506_c0_g2	Tubby-related protein 1	Metazoa; Mammalia; Hominidae	0.01734168	0	33.606	42.296
TRINITY_DN66993_c0_g1	Aryl-hydrocarbon-interacting protein-like 1	Metazoa; Mammalia; Muridae	0.01734168	0	71.596	88.41
TRINITY_DN69921_c4_g1	*No Match*	*No Match*	0.01734168	0	43.663	53.443
TRINITY_DN64267_c0_g1	Homeobox protein otx5	Metazoa; Amphibia; Pipidae	0.017408199	0	52.15	213.716
TRINITY_DN69731_c4_g1	*No Match*	*No Match*	0.017582254	0	59.87	118.393
TRINITY_DN60428_c1_g1	*No Match*	*No Match*	0.017582254	0	36.745	72.921
TRINITY_DN65748_c0_g1	*No Match*	*No Match*	0.017997971	0	49.201	194.97
TRINITY_DN69324_c0_g1	Retinoid isomerohydrolase	Metazoa; Actinopterygii; Cyprinidae	0.017997971	0.306	70.558	125.912
TRINITY_DN65962_c7_g1	Synaptophysin	Metazoa; Actinopterygii; Salmonidae	0.018042754	0	27.572	39.084
TRINITY_DN69432_c3_g1	*No Match*	*No Match*	0.018042754	0	80.994	175.943
TRINITY_DN70483_c4_g1	Uncharacterized protein C2orf71	Metazoa; Mammalia; Hominidae	0.018042754	0	9.434	25.423
TRINITY_DN69018_c0_g1	Complexin 4a	Metazoa; Actinopterygii; Cyprinidae	0.018973677	0	39.397	86.673
TRINITY_DN70729_c1_g1	Uncharacterized protein	Metazoa; Actinopterygii; Cichlidae	0.019351679	0	21.448	25.459
TRINITY_DN71226_c0_g4	Protocadherin-15	Metazoa; Mammalia; Muridae	0.019351679	0	48.236	99.475
TRINITY_DN69742_c4_g1	*No Match*	*No Match*	0.019351679	0	133.937	0.308
TRINITY_DN61880_c0_g1	Uncharacterized protein C2orf71	Metazoa; Mammalia; Hominidae	0.019727983	0	15.955	26.816
TRINITY_DN69731_c8_g1	*No Match*	*No Match*	0.020557426	0	55.135	90.329
TRINITY_DN67289_c4_g1	cGMP-gated cation channel alpha-1	Metazoa; Mammalia; Muridae	0.020557426	0	38.459	36.669
TRINITY_DN68860_c1_g2	Heterogeneous nuclear ribonucleoprotein A0	Metazoa; Actinopterygii; Salmonidae	0.020557426	0	36.231	68.805
TRINITY_DN70733_c0_g3	Phosducin	Metazoa; Mammalia; Canidae	0.02063239	0	42.842	52.502
TRINITY_DN51487_c0_g1	*No Match*	*No Match*	0.02063239	0	166.66	366.706
TRINITY_DN63229_c0_g1	*No Match*	*No Match*	0.02063239	0	133.036	160.825

TRINITY_DN44906_c0_g2	Guanine nucleotide-binding protein G(T) subunit gamma-T1	Metazoa; Mammalia; Bovidae	0.02063239	0	186.006	0.299
TRINITY_DN59372_c0_g1	*No Match*	*No Match*	0.02063239	0	454.503	77.87
TRINITY_DN65557_c0_g2	Cystatin	Metazoa; Actinopterygii; Osmeridae	0.02063239	0	30.963	84.792
TRINITY_DN45143_c0_g1	Elongation factor 1-alpha	Metazoa; Actinopterygii; Tetraodontidae	0.02063239	0.172	55.686	61.585
TRINITY_DN67491_c7_g1	Polysialic acid O-acetyltransferase	Bacteria; Gammaproteobacteria; Enterobacteriaceae	0.020836876	0	33.768	51.515
TRINITY_DN70883_c0_g1	Cadherin-related family member 1	Metazoa; Actinopterygii; Cyprinidae	0.02086276	0	22.35	46.748
TRINITY_DN69654_c0_g4	Amino acid transporter	Metazoa; Actinopterygii; Tetraodontidae	0.022254672	0	8.686	18.339
TRINITY_DN65277_c1_g1	Homeobox protein otx5	Metazoa; Amphibia; Pipidae	0.022254672	1.078	155.223	335.059
TRINITY_DN71055_c0_g1	*No Match*	*No Match*	0.02325171	0	3.725	73.953
TRINITY_DN69731_c7_g1	Interphotoreceptor matrix proteoglycan 2	Metazoa; Aves; Phasianidae	0.023274875	0	34.851	34.154
TRINITY_DN69986_c0_g2	Chromosome 12 SCAF14999	Metazoa; Actinopterygii; Tetraodontidae	0.023284476	0	198.597	126.4
TRINITY_DN64187_c0_g1	Potassium voltage-gated channel subfamily V member 2	Metazoa; Mammalia; Muridae	0.023284476	0	23.099	39.555
TRINITY_DN69290_c0_g1	G-protein-coupled receptor kinase 1a	Metazoa; Actinopterygii; Cyprinidae	0.023419956	0.172	37.106	72.605
TRINITY_DN47315_c0_g2	*No Match*	*No Match*	0.02491745	0	25.038	1998.437
TRINITY_DN39620_c0_g1	*No Match*	*No Match*	0.025230125	0	273.241	183.579
TRINITY_DN65272_c0_g1	Echinoderm microtubule-associated protein-like 1	Metazoa; Mammalia; Hominidae	0.025230125	0	22.088	62.617
TRINITY_DN70616_c1_g1	Synembryn-A	Metazoa; Amphibia; Pipidae	0.025230125	0	33.832	31.557
TRINITY_DN70006_c1_g1	Delta and Notch-like epidermal growth factor-related receptor	Metazoa; Mammalia; Hominidae	0.026162882	0	13.511	29.042
TRINITY_DN38168_c0_g2	*No Match*	*No Match*	0.026162882	0	486.035	575.238
TRINITY_DN69246_c4_g14	Uncharacterized protein	Metazoa; Actinopterygii; Adrianichthyidae	0.02636436	0	727.565	42.17
TRINITY_DN65558_c0_g1	RBP3-like protein-2	Metazoa; Actinopterygii; Osmeridae	0.02636436	0.907	234.675	91.306
TRINITY_DN70781_c1_g2	*No Match*	*No Match*	0.02636436	0	61.882	95.675
TRINITY_DN68675_c0_g2	Excitatory amino acid transporter 5	Metazoa; Mammalia; Hominidae	0.027485367	0	34.111	38.922
TRINITY_DN33844_c0_g2	*No Match*	*No Match*	0.027927168	0	71.271	93.377

TRINITY_DN71102_c3_g4	Photoreceptor outer segment membrane glycoprotein 2	Metazoa; Aves; Phasianidae	0.028222186	0	37.755	35.04
TRINITY_DN70068_c4_g1	*No Match*	*No Match*	0.029218571	0	175.67	102.588
TRINITY_DN46053_c0_g1	*No Match*	*No Match*	0.029253029	0	131.683	264.698
TRINITY_DN69920_c2_g1	Retinal-specific ATP-binding cassette transporter	Metazoa; Mammalia; Hominidae	0.030075402	0	12.302	20.782
TRINITY_DN70319_c4_g11	*No Match*	*No Match*	0.030143298	0.797	96.534	191.668
TRINITY_DN70619_c5_g3	*No Match*	*No Match*	0.030145736	0	420.221	306.894
TRINITY_DN61128_c0_g2	*No Match*	*No Match*	0.030145736	0	113.653	267.258
TRINITY_DN64536_c0_g1	*No Match*	*No Match*	0.030194722	0	13.755	34.923
TRINITY_DN62765_c0_g1	Gamma-crystallin M2	Metazoa; Chondrichthyes; Galeoidea	0.030229089	0	264.511	0
TRINITY_DN70804_c1_g2	*No Match*	*No Match*	0.030555968	0.135	19.247	51.868
TRINITY_DN70844_c1_g5	Gamma-crystallin M2	Metazoa; Actinopterygii; Cyprinidae	0.030733023	0	2070.565	8.622
TRINITY_DN66352_c0_g1	Beta-adducin	Metazoa; Actinopterygii; Salmonidae	0.030733023	0.306	56.272	46.576
TRINITY_DN69100_c0_g2	Leucine-rich repeat, immunoglobulin-like domain and transmembrane	Metazoa; Mammalia; Hominidae	0.030866943	0	21.737	20.99
TRINITY_DN66496_c1_g3	Retinoschisin	Metazoa; Actinopterygii; Tetraodontidae	0.030866943	0.355	53.016	95.694
TRINITY_DN69869_c0_g1	Leucine-rich repeat, immunoglobulin-like domain and transmembrane domain-containing protein 2	Metazoa; Actinopterygii; Cyprinidae	0.031367797	0	31.099	28.933
TRINITY_DN69489_c2_g1	Serine incorporator 4	Metazoa; Mammalia; Muridae	0.031367797	0	18.95	48.141
TRINITY_DN71240_c0_g1	Protein unc-13 homolog B	Metazoa; Mammalia; Hominidae	0.031367797	0	1.939	5.266
TRINITY_DN70594_c2_g1	*No Match*	*No Match*	0.031438803	0	19.725	44.142
TRINITY_DN71195_c1_g1	Chondroitin sulfate proteoglycan 4	Metazoa; Mammalia; Muridae	0.031662629	0.061	15.585	17.416
TRINITY_DN20507_c0_g1	*No Match*	*No Match*	0.031662629	0	443.356	923.904
TRINITY_DN59677_c0_g1	Protein lifeguard 2	Metazoa; Mammalia; Hominidae	0.032194022	0	25.813	20.212
TRINITY_DN69087_c1_g2	Testican-1	Metazoa; Mammalia; Muridae	0.032194022	0	13.006	62.598
TRINITY_DN70932_c0_g1	*No Match*	*No Match*	0.032491142	0	1.948	15.073
TRINITY_DN67036_c2_g1	Potassium voltage-gated channel subfamily B	Metazoa; Mammalia; Bovidae	0.032491142	0	9.272	30.381

	member 2					
TRINITY_DN69849_c3_g1	Retinal-specific ATP-binding cassette transporter	Metazoa; Mammalia; Muridae	0.032541445	0	36.483	37.257
TRINITY_DN70844_c1_g8	Gamma-crystallin M1	Metazoa; Chondrichthyes; Galeoidea	0.032541445	0	766.023	2.606
TRINITY_DN69442_c0_g1	*No Match*	*No Match*	0.032597044	0	57.426	38.949
TRINITY_DN62691_c0_g3	*No Match*	*No Match*	0.033051283	10	1848.968	1049.029
TRINITY_DN70566_c1_g1	*No Match*	*No Match*	0.033051283	0	61.792	14.892
TRINITY_DN70790_c0_g1	LINE-1 reverse transcriptase homolog	Metazoa; Mammalia; Lorisidae	0.033284891	0	21.836	26.111
TRINITY_DN70162_c4_g1	*No Match*	*No Match*	0.03376548	0	47.604	48.774
TRINITY_DN70564_c4_g2	Uncharacterized protein	Metazoa; Actinopterygii; Cyprinidae	0.033874289	0	15.153	144.305
TRINITY_DN55138_c0_g3	Estrogen-related receptor gamma	Metazoa; Mammalia; Muridae	0.0342383	0	23.694	51.778
TRINITY_DN63087_c1_g1	Synaptosomal-associated protein	Metazoa; Actinopterygii; Poeciliidae	0.0342383	0	15.658	31.059
TRINITY_DN70363_c2_g1	Protein phosphatase 1B	Metazoa; Mammalia; Hominidae	0.034496225	0	23.333	24.898
TRINITY_DN38696_c0_g1	*No Match*	*No Match*	0.03532047	0	332.481	401.538
TRINITY_DN69322_c0_g1	Hnf4a protein	Metazoa; Actinopterygii; Cyprinidae	0.037897689	0	6.305	34.018
TRINITY_DN26248_c0_g3	Gamma-crystallin N	Metazoa; Mammalia; Muridae	0.03803773	0	118.776	0
TRINITY_DN69905_c1_g1	Transporter	Metazoa; Actinopterygii; Cichlidae	0.03803773	0	13.15	15.697
TRINITY_DN70147_c1_g1	Hemoglobin subunit alpha-2	Metazoa; Actinopterygii; Nototheniidae	0.038065431	0	896.199	81.345
TRINITY_DN45135_c0_g1	60S ribosomal protein L34	Metazoa; Actinopterygii; Salmonidae	0.038065431	0	176.446	200.579
TRINITY_DN62683_c0_g2	*No Match*	*No Match*	0.038065431	0	99.276	708.189
TRINITY_DN69845_c3_g10	Regulator of G-protein signaling 8	Metazoa; Actinopterygii; Cyprinidae	0.040804505	0	16.018	20.094
TRINITY_DN62683_c0_g3	*No Match*	*No Match*	0.040806451	0	181.551	660.066
TRINITY_DN70638_c0_g1	MAGUK p55 subfamily member 4	Metazoa; Mammalia; Hominidae	0.041377887	0	26.778	28.164
TRINITY_DN68054_c1_g3	Voltage-dependent L-type calcium channel subunit beta-4	Metazoa; Mammalia; Muridae	0.041377887	0	25.056	44.459
TRINITY_DN51404_c0_g2	LINE-1 reverse transcriptase homolog	Metazoa; Mammalia; Lorisidae	0.042569479	0	1.091	14.81
TRINITY_DN66349_c0_g1	Clathrin coat assembly protein AP180	Metazoa; Mammalia; Hominidae	0.042827489	0	15.793	28.924
TRINITY_DN69152_c1_g1	*No Match*	*No Match*	0.042845149	0	93.585	52.791

TRINITY_DN67377_c0_g1	*No Match*	*No Match*	0.042863988	0	35.13	28.228
TRINITY_DN70242_c1_g2	F-env	Viruses; dsDNA viruses, Herpesviridae	0.043916496	0	3.04	32.57
TRINITY_DN69720_c3_g1	Uncharacterized protein	Metazoa; Actinopterygii; Cyprinidae	0.044604518	0	43.924	36.334
TRINITY_DN70031_c0_g2	Retinaldehyde-binding protein 1	Metazoa; Mammalia; Bovidae	0.044632236	0	29.854	55.713
TRINITY_DN60505_c0_g1	Uncharacterized protein	Metazoa; Actinopterygii; Cichlidae	0.044878335	0	40.452	0.072
TRINITY_DN70319_c4_g10	1-acyl-sn-glycerol-3-phosphate acyltransferase gamma	Metazoa; Mammalia; Hominidae	0.046191783	0.699	46.756	102.57
TRINITY_DN71080_c0_g1	*No Match*	*No Match*	0.046780535	0	53.855	147.571
TRINITY_DN71037_c1_g3	LINE-1 reverse transcriptase homolog	Metazoa; Mammalia; Lorisidae	0.047525552	0	15.216	24.835
TRINITY_DN62096_c0_g1	*No Match*	*No Match*	0.047916694	0	70.748	145.562
TRINITY_DN38168_c0_g1	*No Match*	*No Match*	0.04887804	0	402.227	274.306
TRINITY_DN61625_c0_g1	Cytochrome c oxidase subunit NDUFA4	Metazoa; Actinopterygii; Cyprinidae	0.04892844	0	32.47	47.001
TRINITY_DN69982_c0_g2	*No Match*	*No Match*	0.04972214	0	51.482	49.887
TRINITY_DN69058_c1_g3	Sodium/potassium/calcium exchanger 1	Metazoa; Aves; Phasianidae	0.04972214	0	17.155	35.285
TRINITY_DN67983_c0_g1	Pleckstrin homology domain-containing family B member 1	Metazoa; Mammalia; Muridae	0.04972214	0	21.421	63.503

Supplementary Table S2. Retina isoforms significantly up-regulated in *Malacosteus* relative to *Pachystomias*. For each isoform, annotation with respect to the most similar protein sequence hit from BLASTx searches (Protein Identity) and the organism from which this sequence derived (Taxon) are given. For differential expression analyses, the multi-test corrected significance level (FDR) and normalized expression values per individual (PM1, MN1, MN2) for each isoform are also given.

Isoform ID	Protein Identity	Taxon	FDR	AM1	MN1	MN2
TRINITY_DN65229_c0_g1_i1	Guanine nucleotide-binding protein G(t) subunit alpha-1	Metazoa; Mammalia; Muridae	0.000106274	0	596.482	1093.152
TRINITY_DN50791_c0_g1_i1	Rhodopsin	Metazoa; Actinopterygii; Stomiidae	0.000106274	1.213	8193.735	9932.388
TRINITY_DN60692_c0_g1_i4	Cytochrome c oxidase subunit 1	Metazoa; Actinopterygii; Salmonidae	0.000165384	0	137.725	459.121
TRINITY_DN62339_c0_g2_i1	*No Match*	*No Match*	0.000165384	3.798	36700.641	67444.832
TRINITY_DN38768_c0_g3_i1	Retinal rod rhodopsin-sensitive cGMP 3',5'-cyclic phosphodiesterase subunit	Metazoa; Mammalia; Bovidae	0.000165384	0	1246.14	2739.163
TRINITY_DN44906_c0_g1_i1	Guanine nucleotide-binding protein subunit gamma	Metazoa; Actinopterygii; Gasterosteidae	0.000174257	0	1214.613	2315.939
TRINITY_DN34250_c0_g1_i1	NADH-ubiquinone oxidoreductase chain 5	Metazoa; Actinopterygii; Retropinnidae	0.000217418	0	338.876	359.5
TRINITY_DN62973_c1_g2_i1	*No Match*	*No Match*	0.00024554	0	1728.933	1558.328
TRINITY_DN60692_c0_g1_i2	Cytochrome c oxidase subunit 1	Metazoa; Actinopterygii; Salmonidae	0.000323699	0.053	334.819	200.063
TRINITY_DN39938_c0_g2_i1	*No Match*	*No Match*	0.000337359	2.468	14317.609	5495.267
TRINITY_DN60692_c0_g1_i3	Cytochrome c oxidase subunit 1	Metazoa; Actinopterygii; Salmonidae	0.000345224	0	63.315	193.37
TRINITY_DN48042_c0_g1_i1	Cytochrome c oxidase subunit 3	Metazoa; Actinopterygii; Stomiidae	0.000351328	0.17	646.484	774.562
TRINITY_DN60692_c0_g1_i1	Cytochrome c oxidase subunit 1	Metazoa; Actinopterygii; Salmonidae	0.000389673	0	142.181	90.579
TRINITY_DN69457_c2_g1_i1	Riboflavin-binding protein	Metazoa; Actinopterygii; Salmonidae	0.000433563	0	331.3	442.555
TRINITY_DN38768_c0_g2_i1	*No Match*	*No Match*	0.000459045	0	1128.784	2284.497
TRINITY_DN54646_c0_g1_i1	Guanine nucleotide-binding protein G(I)/G(S)/G(T) subunit beta-1	Metazoa; Actinopterygii; Cyprinidae	0.000486197	0	134.364	259.656
TRINITY_DN69731_c6_g1_i3	Interphotoreceptor matrix proteoglycan 2	Metazoa; Aves; Phasianidae	0.000577596	0	65.748	114.092
TRINITY_DN45697_c1_g1_i1	Peripherin-2	Metazoa; Aves; Phasianidae	0.000884783	0	278.745	522.552
TRINITY_DN57337_c0_g1_i1	Peripherin-2	Metazoa; Mammalia; Canidae	0.000996888	0	174.061	353.232

TRINITY_DN57898_c0_g1_i1	*No Match*	*No Match*	0.001681784	1.713	3130.502	5004.886
TRINITY_DN7106_c0_g1_i1	*No Match*	*No Match*	0.001774173	0	85.717	195.121
TRINITY_DN69921_c3_g2_i2	Rod cGMP-specific 3',5'-cyclic phosphodiesterase subunit alpha	Metazoa; Actinopterygii; Ictaluridae	0.00195564	0	98.816	166.08
TRINITY_DN70660_c2_g1_i1	Prominin-1-A	Metazoa; Actinopterygii; Cyprinidae	0.002410171	0	31.268	92.138
TRINITY_DN69139_c0_g1_i1	Hemoglobin subunit beta-2	Metazoa; Actinopterygii; Gadidae	0.00283629	0	1134.28	72.007
TRINITY_DN69026_c5_g1_i1	Retinol dehydrogenase 3	Metazoa; Mammalia; Muridae	0.002859461	0	76.712	118.507
TRINITY_DN62691_c0_g3_i2	*No Match*	*No Match*	0.002915745	0	475.839	691.963
TRINITY_DN54320_c0_g1_i1	Creatine kinase, mitochondrial 2	Metazoa; Actinopterygii; Salmonidae	0.002915745	0	202.154	114.649
TRINITY_DN65214_c0_g1_i1	*No Match*	*No Match*	0.003370432	0	3465.906	102.264
TRINITY_DN59359_c0_g1_i1	Type-4 ice-structuring protein LS-12	Metazoa; Actinopterygii; Cottidae	0.003407026	0	328.719	316.97
TRINITY_DN69921_c3_g2_i4	Rod cGMP-specific 3',5'-cyclic phosphodiesterase subunit beta	Metazoa; Mammalia; Bovidae	0.003675311	0	91.881	107.641
TRINITY_DN70278_c1_g27_i4	Interphotoreceptor matrix proteoglycan 1	Metazoa; Aves; Phasianidae	0.004041715	0	28.937	56.656
TRINITY_DN64536_c0_g2_i1	*No Match*	*No Match*	0.004249844	0	178.295	321.172
TRINITY_DN53485_c0_g1_i1	NADH-ubiquinone oxidoreductase chain 4	Metazoa; Actinopterygii; Salmonidae	0.004551072	0	121.961	131.59
TRINITY_DN70830_c3_g2_i7	ATP-binding cassette sub-family A member 1	Metazoa; Mammalia; Hominidae	0.004551072	0	58.59	58.114
TRINITY_DN70844_c1_g14_i1	Gamma-crystallin M2	Metazoa; Actinopterygii; Cyprinidae	0.005296359	0	932.052	0
TRINITY_DN58067_c0_g2_i1	Gamma-crystallin M3	Metazoa; Actinopterygii; Cyprinidae	0.005296359	0	1327.903	3.129
TRINITY_DN63039_c0_g1_i1	Complexin-4	Metazoa; Mammalia; Muridae	0.005828635	0	89.356	191.871
TRINITY_DN65797_c0_g1_i2	RNA-binding protein Musashi homolog 1	Metazoa; Mammalia; Hominidae	0.006069184	0	47.672	71.4
TRINITY_DN64954_c0_g1_i1	*No Match*	*No Match*	0.006069184	0	199.48	364.502
TRINITY_DN106325_c0_g1_i1	*No Match*	*No Match*	0.006362212	0	2382.63	3.868
TRINITY_DN67956_c0_g1_i4	*No Match*	*No Match*	0.006362212	0	296.588	385.008
TRINITY_DN45697_c1_g2_i1	Peripherin-2	Metazoa; Aves; Phasianidae	0.006362212	0	48.433	166.211
TRINITY_DN70830_c3_g2_i6	ATP-binding cassette sub-family A member 1	Metazoa; Mammalia; Hominidae	0.006926368	0	41.61	24.171
TRINITY_DN63927_c0_g1_i1	Retinol dehydrogenase 7	Metazoa; Mammalia; Muridae	0.007081289	0	168.333	124.329
TRINITY_DN71136_c1_g2_i1	Calcium-transporting ATPase	Metazoa; Actinopterygii; Gasterosteida	0.007081289	0	16.117	31.422
TRINITY_DN67660_c0_g1_i1	Protein LBH	Metazoa; Mammalia; Bovidae	0.007152076	0	40.756	115.378
TRINITY_DN64954_c1_g1_i1	*No Match*	*No Match*	0.007152076	0	353.656	350.599

TRINITY_DN62400_c0_g6_i1	*No Match*	*No Match*	0.007786225	0	1608.086	1.681
TRINITY_DN79162_c0_g1_i1	Synaptotagmin-2	Metazoa; Mammalia; Muridae	0.007786225	0	81.808	131.671
TRINITY_DN70717_c0_g1_i4	Sterile alpha motif domain-containing protein 7	Metazoa; Mammalia; Hominidae	0.008330354	0	15.634	111.732
TRINITY_DN67162_c3_g1_i1	Voltage-dependent L-type calcium channel subunit alpha-1F	Metazoa; Mammalia; Hominidae	0.008330354	0	17.101	32.201
TRINITY_DN71259_c1_g1_i2	Polyprotein	Viruses; Retro-transcribing viruses; Retroviridae;	0.008528019	0	0.743	24.323
TRINITY_DN70278_c1_g27_i5	Interphotoreceptor matrix proteoglycan 1	Metazoa; Aves; Phasianidae	0.008528019	0	28.77	30.925
TRINITY_DN3114_c0_g1_i1	Diamine acetyltransferase 1	Metazoa; Actinopterygii; Salmonidae	0.008528019	0	87.899	134.557
TRINITY_DN65876_c0_g1_i1	*No Match*	Metazoa; Actinopterygii; Poeciliidae	0.008528019	0	100.924	39.229
TRINITY_DN61263_c0_g1_i1	Guanylyl cyclase-activating protein 2	Metazoa; Actinopterygii; Cyprinidae	0.008528019	0	64.531	65.82
TRINITY_DN70565_c0_g2_i2	S-arrestin	Metazoa; Mammalia; Bovidae	0.009082035	0	99.949	18.622
TRINITY_DN67838_c0_g2_i1	SEC14-like protein 2	Metazoa; Mammalia; Bovidae	0.009117531	0	19.942	56.899
TRINITY_DN69324_c0_g1_i1	Retinoid isomerohydrolase	Metazoa; Actinopterygii; Cyprinidae	0.009185288	0	40.858	61.628
TRINITY_DN65277_c0_g1_i1	Homeobox protein otx5-A	Metazoa; Amphibia; Pipidae	0.009334147	0.138	56.807	124.694
TRINITY_DN65306_c1_g1_i2	Beta-crystallin A1	Metazoa; Amphibia; Ranidae	0.011373512	0	253.02	0
TRINITY_DN58894_c0_g1_i1	Vesicle-associated membrane protein 2	Metazoa; Mammalia; Muridae	0.011443614	0	36.597	41.041
TRINITY_DN70830_c3_g2_i4	ATP-binding cassette sub-family A member 1	Metazoa; Mammalia; Hominidae	0.011443614	0	19.004	27.624
TRINITY_DN67956_c0_g1_i2	*No Match*	*No Match*	0.011563803	0	260.372	188.286
TRINITY_DN67846_c10_g4_i2	Uncharacterized protein	Metazoa; Actinopterygii; Cyprinidae	0.011563803	0	53.939	122.598
TRINITY_DN66896_c0_g1_i1	NADH-ubiquinone oxidoreductase chain 5	Metazoa; Actinopterygii; Retropinnidae	0.012754176	0	14.77	27.655
TRINITY_DN38448_c0_g1_i1	*No Match*	*No Match*	0.012754176	0	251.107	0.435
TRINITY_DN69731_c6_g1_i2	Interphotoreceptor matrix proteoglycan 2	Metazoa; Aves; Phasianidae	0.012865507	0	1.133	30.581
TRINITY_DN57610_c0_g1_i2	Glucose-6-phosphate 1-dehydrogenase	Metazoa; Actinopterygii; Salmonidae	0.012865507	0	27.749	46.186
TRINITY_DN70996_c0_g6_i1	Syntaxin-binding protein 1	Metazoa; Mammalia; Muridae	0.013010012	0	22.792	49.406
TRINITY_DN69246_c4_g4_i1	Uncharacterized protein	Metazoa; Actinopterygii; Adrianichthyidae	0.013040934	0	1141.689	7.331
TRINITY_DN53171_c0_g1_i1	Protein lifeguard 2	Metazoa; Mammalia; Hominidae	0.01308966	0	69.693	137.625
TRINITY_DN70844_c1_g14_i4	Gamma-crystallin M1	Metazoa; Chondrichthyes; Hemiscylliidae	0.013460603	4.266	6311.833	10.673
TRINITY_DN70844_c1_g14_i8	*No Match*	*No Match*	0.013460603	0	1553.247	1.59
TRINITY_DN58067_c0_g1_i1	Gamma-crystallin M3	Metazoa; Actinopterygii; Cyprinidae	0.014532394	0	683.313	1.448

TRINITY_DN69731_c4_g1_i1	*No Match*	*No Match*	0.014532394	0	61.626	132.511
TRINITY_DN61070_c0_g1_i2	Beta-crystallin B1	Metazoa; Mammalia; Suidae	0.014669308	2.734	1529.592	4.861
TRINITY_DN70417_c2_g1_i2	Putative ATP-dependent RNA helicase an3	Metazoa; Amphibia; Pipidae	0.015080997	0	0	34.885
TRINITY_DN68489_c0_g1_i1	ATPase, Na+/K+ transporting, alpha 3a polypeptide	Metazoa; Actinopterygii; Cyprinidae	0.015080997	0	1.374	52.646
TRINITY_DN70958_c5_g1_i2	Protein piccolo	Metazoa; Mammalia; Muridae	0.015176303	0	5.71	11.716
TRINITY_DN65962_c7_g1_i1	Synaptophysin	Metazoa; Actinopterygii; Salmonidae	0.015176303	0	28.38	43.745
TRINITY_DN69432_c3_g1_i1	*No Match*	*No Match*	0.015176303	0	83.368	196.924
TRINITY_DN71056_c0_g1_i3	CD36 antigen	Metazoa; Actinopterygii; Oplegnathidae	0.015382124	0	4.976	36.637
TRINITY_DN69731_c6_g1_i1	Interphotoreceptor matrix proteoglycan 2	Metazoa; Aves; Phasianidae	0.015926681	0	7.863	18.936
TRINITY_DN65277_c1_g1_i2	Homeobox protein otx5	Metazoa; Amphibia; Pipidae	0.016272288	0	0	171.102
TRINITY_DN61880_c0_g1_i1	Uncharacterized protein C2orf71	Metazoa; Mammalia; Hominidae	0.017003039	0	16.423	30.014
TRINITY_DN70830_c3_g2_i1	ATP-binding cassette sub-family A member 1	Metazoa; Mammalia; Muridae	0.01740266	0	23.525	11.473
TRINITY_DN63747_c1_g2_i3	Beta-crystallin S	Metazoa; Actinopterygii; Cyprinidae	0.01740266	0	314.924	0.466
TRINITY_DN66993_c0_g1_i1	Aryl-hydrocarbon-interacting protein-like 1	Metazoa; Mammalia; Muridae	0.017856568	0	73.694	74.093
TRINITY_DN70849_c3_g1_i3	Retinol-binding protein I, cellular	Metazoa; Actinopterygii; Salmonidae	0.017878414	0	28.91	30.237
TRINITY_DN70469_c4_g1_i1	Monocarboxylate transporter 1	Metazoa; Mammalia; Cricetidae	0.017878414	0	9.135	27.999
TRINITY_DN68860_c1_g2_i1	Heterogeneous nuclear ribonucleoprotein A0	Metazoa; Actinopterygii; Salmonidae	0.017878414	0	37.293	77.01
TRINITY_DN67290_c0_g4_i1	*No Match*	*No Match*	0.017878414	0	51.933	36.525
TRINITY_DN65306_c0_g1_i1	Beta-crystallin A3	Metazoa; Mammalia; Muridae	0.017878414	0	180.978	0
TRINITY_DN70733_c0_g3_i1	Phosducin	Metazoa; Mammalia; Canidae	0.018463211	0	44.098	58.762
TRINITY_DN70793_c2_g1_i6	Heat shock 90kDa protein 1 beta isoform b	Metazoa; Actinopterygii; Salmonidae	0.018484133	0	12.682	47.259
TRINITY_DN63229_c0_g1_i1	*No Match*	*No Match*	0.018513944	0	136.935	180.003
TRINITY_DN45143_c0_g1_i1	Elongation factor 1-alpha	Metazoa; Actinopterygii; Tetraodontidae	0.018793877	0.149	57.318	68.929
TRINITY_DN67613_c0_g1_i1	*No Match*	*No Match*	0.01896061	0	23.674	93.88
TRINITY_DN59372_c0_g1_i1	*No Match*	*No Match*	0.019460029	0	467.827	87.156
TRINITY_DN44906_c0_g2_i1	Guanine nucleotide-binding protein G(T) subunit gamma-T1	Metazoa; Mammalia; Bovidae	0.019601124	0	191.459	0.334
TRINITY_DN58487_c0_g1_i1	Ferritin	Metazoa; Actinopterygii; Salmonidae	0.020131329	0	27.016	122.426
TRINITY_DN65558_c0_g1_i1	RBP3-like protein-2	Metazoa; Actinopterygii; Osmeridae	0.020903884	0.787	241.554	102.193
TRINITY_DN70953_c2_g2_i6	Elongation factor 2	Metazoa; Mammalia; Muridae	0.020982858	0	51.024	0

TRINITY_DN64187_c0_g1_i1	Potassium voltage-gated channel subfamily V member 2	Metazoa; Mammalia; Muridae	0.02127667	0	23.776	44.272
TRINITY_DN69290_c0_g1_i1	G-protein-coupled receptor kinase 1a	Metazoa; Actinopterygii; Cyprinidae	0.021303966	0.149	38.193	81.263
TRINITY_DN47315_c0_g2_i1	*No Match*	*No Match*	0.021374739	0	25.772	2236.742
TRINITY_DN70642_c0_g1_i4	Oxygen-regulated protein 1	Metazoa; Mammalia; Bovidae	0.021374739	0	11.224	9.65
TRINITY_DN69097_c0_g1_i2	Neuronal pentraxin-1	Metazoa; Mammalia; Muridae	0.021393116	0	21.195	28.131
TRINITY_DN69762_c1_g1_i1	Complement C4	Metazoa; Mammalia; Muridae	0.021393116	0	11.818	12.526
TRINITY_DN66496_c1_g3_i1	Retinoschisin	Metazoa; Actinopterygii; Tetraodontidae	0.022625392	0.308	54.57	107.105
TRINITY_DN39620_c0_g1_i1	*No Match*	*No Match*	0.022625392	0	281.251	205.47
TRINITY_DN65557_c0_g2_i2	Cystatin	Metazoa; Actinopterygii; Osmeridae	0.022625392	0	29.699	79.258
TRINITY_DN38168_c0_g2_i1	*No Match*	*No Match*	0.022926921	0	500.283	643.833
TRINITY_DN70308_c3_g1_i1	L-lactate dehydrogenase	Metazoa; Actinopterygii; Macrouridae	0.023112148	0	35.288	26.713
TRINITY_DN70278_c1_g27_i1	Interphotoreceptor matrix proteoglycan 1	Metazoa; Aves; Phasianidae	0.023112148	0	11.335	9.964
TRINITY_DN69742_c4_g1_i1	*No Match*	*No Match*	0.023653044	0	108.704	0.172
TRINITY_DN69281_c1_g1_i6	Guanylate cyclase	Metazoa; Actinopterygii; Cichlidae	0.024060076	0	13.155	21.852
TRINITY_DN70319_c4_g11_i2	1-acyl-sn-glycerol-3-phosphate acyltransferase gamma	Metazoa; Mammalia; Hominidae	0.024458305	0.691	99.364	159.001
TRINITY_DN33844_c0_g2_i1	*No Match*	*No Match*	0.024461727	0	73.36	104.512
TRINITY_DN69299_c1_g1_i1	Poly(rC)-binding protein 3	Metazoa; Mammalia; Hominidae	0.024772938	0	20.229	22.693
TRINITY_DN71102_c3_g4_i1	Photoreceptor outer segment membrane glycoprotein 2	Metazoa; Aves; Phasianidae	0.024772938	0	38.862	39.219
TRINITY_DN65876_c0_g1_i2	*No Match*	*No Match*	0.024772938	0	17.946	54.509
TRINITY_DN70883_c0_g1_i1	Cadherin-related family member 1	Metazoa; Actinopterygii; Cyprinidae	0.024780767	0	17.565	37.538
TRINITY_DN46053_c0_g1_i1	*No Match*	*No Match*	0.024780767	0	135.543	296.262
TRINITY_DN69018_c0_g1_i3	Complexin 4a	Metazoa; Actinopterygii; Cyprinidae	0.025716439	0	15.068	53.203
TRINITY_DN69921_c4_g1_i2	*No Match*	*No Match*	0.025716439	0	32.605	29.103
TRINITY_DN70804_c1_g2_i1	*No Match*	*No Match*	0.026198867	0.117	19.812	58.053
TRINITY_DN67159_c0_g1_i2	L-threonine ammonia-lyase	Bacteria; Deltaproteobacteria; Geobacteraceae	0.026858343	0	0	36.161
TRINITY_DN68850_c1_g3_i1	Basic leucine zipper and W2 domain-containing protein 2	Metazoa; Actinopterygii; Cyprinidae	0.02773233	0	42.603	40.12

TRINITY_DN70594_c2_g1_i1	*No Match*	*No Match*	0.02773233	0	20.304	49.406
TRINITY_DN68980_c1_g3_i2	Vesicle-fusing ATPase	Metazoa; Actinopterygii; Salmonidae	0.02773233	0	13.935	22.531
TRINITY_DN20507_c0_g1_i1	*No Match*	*No Match*	0.028307419	0	456.352	1034.076
TRINITY_DN71195_c1_g1_i1	Chondroitin sulfate proteoglycan 4	Metazoa; Mammalia; Muridae	0.028327283	0.053	16.042	19.493
TRINITY_DN70844_c1_g5_i1	Gamma-crystallin M2	Metazoa; Actinopterygii; Cyprinidae	0.028327283	0	2131.263	9.65
TRINITY_DN61128_c0_g2_i2	*No Match*	*No Match*	0.028327283	0	92.336	266.035
TRINITY_DN70793_c2_g1_i1	Heat shock protein HSP 90-alpha 1	Metazoa; Actinopterygii; Salmonidae	0.02871751	0	28.761	47.36
TRINITY_DN59677_c0_g1_i1	Protein lifeguard 2	Metazoa; Mammalia; Hominidae	0.0290048	0	26.57	22.622
TRINITY_DN63626_c0_g1_i1	Scavenger receptor class B type I	Metazoa; Actinopterygii; Salmonidae	0.029047955	0	13.851	27.361
TRINITY_DN65748_c0_g1_i2	*No Match*	*No Match*	0.030002645	0	42.696	120.421
TRINITY_DN69281_c1_g1_i4	Guanylate cyclase	Metazoa; Actinopterygii; Lepisosteidae	0.030002645	0	30.924	20.587
TRINITY_DN69324_c0_g1_i2	Retinoid isomerohydrolase	Metazoa; Actinopterygii; Cyprinidae	0.030002645	0.266	31.76	79.298
TRINITY_DN69869_c0_g1_i1	Leucine-rich repeat, immunoglobulin-like domain and transmembrane domain-containing protein 2	Metazoa; Actinopterygii; Cyprinidae	0.030153268	0	17.444	31.584
TRINITY_DN70513_c4_g17_i3	*No Match*	*No Match*	0.030206302	0	141.299	188.742
TRINITY_DN71226_c0_g4_i3	Protocadherin-15	Metazoa; Aves; Phasianidae	0.030206302	0	19.951	26.834
TRINITY_DN70162_c4_g1_i1	*No Match*	*No Match*	0.030206302	0	49	54.59
TRINITY_DN70844_c1_g8_i1	Gamma-crystallin M1	Metazoa; Aves; Phasianidae	0.030206302	0	788.479	2.916
TRINITY_DN63087_c1_g1_i1	Synaptosomal-associated protein	Metazoa; Actinopterygii; Poeciliidae	0.030267114	0	16.117	34.763
TRINITY_DN70513_c4_g17_i1	*No Match*	*No Match*	0.030267114	0	132.535	145.209
TRINITY_DN38696_c0_g1_i1	*No Match*	*No Match*	0.031597206	0	342.227	449.42
TRINITY_DN70171_c2_g3_i2	DNA-directed RNA polymerase	Metazoa; Actinopterygii; Cyprinidae	0.031949726	0	2.581	19.068
TRINITY_DN65277_c1_g1_i1	Homeobox protein otx5	Metazoa; Amphibia; Pipidae	0.033037106	0.936	159.773	203.911
TRINITY_DN69394_c0_g1_i2	Ubiquitin-like modifier activating enzyme 1	Metazoa; Actinopterygii; Cyprinidae	0.033663263	0	11.308	13.529
TRINITY_DN69905_c1_g1_i1	Transporter	Metazoa; Actinopterygii; Cichlidae	0.033663263	0	13.536	17.569
TRINITY_DN70513_c4_g17_i4	*No Match*	*No Match*	0.03380606	0	210.76	208.984
TRINITY_DN70483_c4_g1_i1	Uncharacterized protein C2orf71	Metazoa; Mammalia; Hominidae	0.033811013	0	4.586	12.597
TRINITY_DN45135_c0_g1_i1	60S ribosomal protein L34	Metazoa; Actinopterygii; Salmonidae	0.033866898	0	181.618	224.497
TRINITY_DN55138_c0_g3_i2	Estrogen-related receptor gamma	Metazoa; Mammalia; Muridae	0.033866898	0	24.388	47.745

TRINITY_DN69920_c2_g1_i2	Retinal-specific ATP-binding cassette transporter	Metazoa; Mammalia; Hominidae	0.033866898	0	12.663	16.415
TRINITY_DN70729_c1_g1_i1	Protein 4.1	Metazoa; Mammalia; Canidae	0.033866898	0	9.841	14.561
TRINITY_DN60428_c1_g1_i2	*No Match*	*No Match*	0.033866898	0	30.395	32.961
TRINITY_DN70790_c0_g1_i1	LINE-1 reverse transcriptase homolog	Metazoa; Mammalia; Lorisidae	0.033877879	0	20.044	21.275
TRINITY_DN26248_c0_g3_i1	Gamma-crystallin N	Metazoa; Mammalia; Muridae	0.034477731	0	122.258	0
TRINITY_DN62683_c0_g3_i1	*No Match*	*No Match*	0.034507291	0	186.873	738.776
TRINITY_DN62765_c0_g1_i3	Gamma-crystallin M2	Metazoa; Chondrichthyes; Hemiscylliidae	0.034507291	0	123.567	0
TRINITY_DN51487_c0_g1_i2	*No Match*	*No Match*	0.034670189	0	169.326	188.6
TRINITY_DN70147_c1_g1_i1	Hemoglobin subunit alpha-2	Metazoa; Actinopterygii; Nototheniidae	0.034687165	0	922.471	91.045
TRINITY_DN51404_c0_g2_i1	LINE-1 reverse transcriptase homolog	Metazoa; Mammalia; Lorisidae	0.035609553	0	1.123	16.577
TRINITY_DN66352_c0_g1_i5	Beta-adducin	Metazoa; Actinopterygii; Salmonidae	0.036434353	0	17.853	32.039
TRINITY_DN68935_c0_g1_i2	Pyruvate carboxylase	Metazoa; Actinopterygii; Cyprinidae	0.036643284	0	5.942	11.098
TRINITY_DN70849_c3_g1_i1	Retinol-binding protein I, cellular	Metazoa; Mammalia; Hominidae	0.036643284	0	40.505	45.811
TRINITY_DN66349_c0_g1_i1	Clathrin coat assembly protein AP180	Metazoa; Mammalia; Hominidae	0.037157123	0	16.256	32.373
TRINITY_DN57024_c0_g1_i2	EGF-like repeat and discoidin I-like domain-containing protein 3	Metazoa; Mammalia; Hominidae	0.038191096	0	20.573	48.373
TRINITY_DN68529_c1_g1_i5	Probable ATP-dependent RNA helicase DDX6	Metazoa; Aves; Phasianidae	0.038950291	0	5.19	11.848
TRINITY_DN70031_c0_g2_i1	Retinaldehyde-binding protein 1	Metazoa; Mammalia; Bovidae	0.039162356	0	30.729	62.357
TRINITY_DN58241_c0_g1_i1	*No Match*	*No Match*	0.039198615	0	102.094	61.618
TRINITY_DN70616_c1_g1_i2	Synembryn-A	Metazoa; Amphibia; Pipidae	0.039415015	0	31.5	12.374
TRINITY_DN62683_c0_g2_i2	*No Match*	*No Match*	0.03949289	0	102.186	727.931
TRINITY_DN67289_c4_g1_i2	Cyclic nucleotide-gated channel rod photoreceptor subunit alpha	Metazoa; Aves; Phasianidae	0.0397151	0	13.443	28.515
TRINITY_DN70660_c2_g1_i2	Prominin-1-A	Metazoa; Actinopterygii; Cyprinidae	0.040149253	0.096	14.501	29.538
TRINITY_DN70872_c2_g3_i2	Putative dystrophin	Metazoa; Actinopterygii; Tetraodontidae	0.040463864	0	1.615	13.842
TRINITY_DN69654_c0_g4_i2	Amino acid transporter	Metazoa; Actinopterygii; Cichlidae	0.040463864	0	6.972	10.4
TRINITY_DN70573_c4_g4_i2	Protein furry homolog	Metazoa; Mammalia; Hominidae	0.040463864	0	5.227	22.956
TRINITY_DN69850_c1_g9_i2	Serine/threonine-protein phosphatase 2A 55 kDa regulatory subunit B	Metazoa; Actinopterygii; Cyprinidae	0.040463864	0	11.651	24.556

TRINITY_DN62096_c0_g1_i1	*No Match*	*No Match*	0.040463864	0	72.822	162.92
TRINITY_DN68572_c0_g1_i2	Lengsin	Metazoa; Mammalia; Canidae	0.040463864	0	40.338	0
TRINITY_DN60428_c1_g1_i1	*No Match*	*No Match*	0.040463864	0	7.427	48.666
TRINITY_DN69281_c0_g1_i2	Retinal guanylyl cyclase 2	Metazoa; Mammalia; Bovidae	0.040463864	0	16.534	18.328
TRINITY_DN70781_c1_g2_i1	*No Match*	*No Match*	0.040463864	0	37.636	75.227
TRINITY_DN70844_c1_g14_i5	Gamma-crystallin M2	Metazoa; Actinopterygii; Cyprinidae	0.040463864	3.17	1029.328	2.309
TRINITY_DN69070_c0_g1_i3	Importin subunit alpha	Metazoa; Mammalia; Cebidae	0.040463864	0	7.306	17.103
TRINITY_DN69246_c4_g14_i1	Uncharacterized protein	Metazoa; Actinopterygii; Adrianichthyidae	0.040463864	0	528.515	4.263
TRINITY_DN69559_c2_g1_i6	*No Match*	*No Match*	0.040465031	0	27.693	8.344
TRINITY_DN70736_c3_g2_i11	Voltage-dependent calcium channel subunit alpha-2/delta-4	Metazoa; Mammalia; Muridae	0.040716599	0	8.244	19.402
TRINITY_DN61625_c0_g1_i1	NADH dehydrogenase (Ubiquinone) 1 alpha subcomplex 4, like	Metazoa; Actinopterygii; Cyprinidae	0.040897749	0	33.422	52.606
TRINITY_DN70736_c3_g2_i2	Voltage-dependent calcium channel subunit alpha-2/delta-4	Metazoa; Mammalia; Muridae	0.040897749	0	15.337	19.108
TRINITY_DN71240_c0_g1_i1	Protein unc-13 homolog B	Metazoa; Mammalia; Hominidae	0.040897749	0	1.532	4.263
TRINITY_DN69215_c6_g3_i2	Nuclear receptor ROR-beta	Metazoa; Mammalia; Hominidae	0.041152071	0	9.293	13.316
TRINITY_DN70053_c3_g2_i1	Muscleblind-like protein 2a	Metazoa; Actinopterygii; Tetraodontidae	0.041152071	0	26.988	135.215
TRINITY_DN38168_c0_g1_i1	*No Match*	*No Match*	0.041152071	0	414.019	307.016
TRINITY_DN67491_c7_g1_i2	Uncharacterized protein	Metazoa; Actinopterygii; Lepisosteidae	0.041827718	0	6.656	38.459
TRINITY_DN68754_c1_g1_i1	Amyloid-like protein 2	Metazoa; Mammalia; Hominidae	0.041827718	0	1.402	25.558
TRINITY_DN69921_c3_g2_i1	Rod cGMP-specific 3',5'-cyclic phosphodiesterase subunit alpha	Metazoa; Actinopterygii; Ictaluridae	0.041827718	0	16.581	16.658
TRINITY_DN70896_c0_g1_i9	Photoreceptor-specific nuclear receptor	Metazoa; Mammalia; Bovidae	0.041898803	0	8.634	28.525
TRINITY_DN69982_c0_g2_i1	*No Match*	*No Match*	0.041970841	0	43.68	55.836
TRINITY_DN69297_c0_g1_i1	LOC560210 protein	Metazoa; Actinopterygii; Cyprinidae	0.042367759	0.543	0.446	151.893
TRINITY_DN70041_c1_g1_i1	Cathepsin F	Metazoa; Actinopterygii; Salmonidae	0.042401928	0	31.769	24.07
TRINITY_DN64536_c0_g2_i2	*No Match*	*No Match*	0.042848002	0	10.212	81.101
TRINITY_DN69559_c2_g1_i3	*No Match*	*No Match*	0.042848002	0	2.618	32.343
TRINITY_DN70471_c1_g5_i8	Inosine-5'-monophosphate dehydrogenase	Metazoa; Actinopterygii; Salmonidae	0.042848002	0	12.542	27.827

TRINITY_DN59065_c0_g1_i1	*No Match*	*No Match*	0.04357932	0	13.675	18.541
TRINITY_DN71166_c0_g1_i1	Androgen receptor	Metazoa; Mammalia; Leporidae	0.043884219	0	9.748	9.124
TRINITY_DN70513_c4_g13_i1	*No Match*	*No Match*	0.044193483	0	298.705	319.451
TRINITY_DN70375_c0_g1_i1	Follistatin-related protein 5	Metazoa; Mammalia; Muridae	0.0447622	0.298	27.118	54.276
TRINITY_DN70319_c4_g10_i2	1-acyl-sn-glycerol-3-phosphate acyltransferase gamma	Metazoa; Mammalia; Hominidae	0.044779865	0	24.249	54.529
TRINITY_DN62566_c1_g1_i1	Transposon Ty3-G Gag-Pol polyprotein	Fungi; Saccharomycetes; Saccharomycetaceae	0.044779865	0	0.566	11.24
TRINITY_DN60505_c0_g1_i2	SPARC	Metazoa; Aves; Phasianidae	0.044779865	0	37.284	0.081
TRINITY_DN68980_c1_g3_i1	Vesicle-fusing ATPase	Metazoa; Actinopterygii; Salmonidae	0.045062268	0	4.419	21.062
TRINITY_DN70513_c3_g2_i1	*No Match*	*No Match*	0.045062268	0	320.392	250.583
TRINITY_DN70483_c4_g1_i2	Uncharacterized protein C2orf71	Metazoa; Mammalia; Hominidae	0.045158588	0	5.134	15.868
TRINITY_DN55272_c0_g1_i1	RNA-directed DNA polymerase from mobile element jockey	Metazoa; Insecta; Drosophilidae	0.045828549	0	28.288	20.718
TRINITY_DN70006_c1_g1_i1	Delta and Notch-like epidermal growth factor-related receptor	Metazoa; Mammalia; Hominidae	0.046379758	0	5.273	19.655
TRINITY_DN70932_c0_g1_i3	*No Match*	*No Match*	0.046485943	0	1.355	10.116
TRINITY_DN59240_c0_g4_i1	Prothymosin alpha-A	Metazoa; Actinopterygii; Cyprinidae	0.04684	0	74.131	107.115
TRINITY_DN69968_c2_g3_i3	Transcriptional regulator ATRX	Metazoa; Mammalia; Hominidae	0.047199277	0	6.536	9.741
TRINITY_DN35711_c0_g1_i1	*No Match*	*No Match*	0.047311224	0	41.87	95.672
TRINITY_DN70519_c4_g1_i4	Protein disulfide-isomerase TMX3	Metazoa; Mammalia; Hominidae	0.047790599	0	8.578	7.22
TRINITY_DN67290_c0_g2_i1	Glycerol-3-phosphate acyltransferase 1, mitochondrial	Metazoa; Mammalia; Muridae	0.047790599	0.287	37.803	34.763
TRINITY_DN65272_c0_g1_i2	CNG-modulin	Metazoa; Actinopterygii; Moronidae	0.048236015	0	18.985	32.272
TRINITY_DN55404_c0_g1_i1	*No Match*	*No Match*	0.048236015	0	37.85	116.411
TRINITY_DN68364_c0_g1_i1	Retinal-specific ATP-binding cassette transporter	Metazoa; Mammalia; Muridae	0.048236015	0	16.423	24.293
TRINITY_DN67613_c0_g1_i2	*No Match*	*No Match*	0.048236015	0	2.794	78.62
TRINITY_DN56658_c0_g1_i1	*No Match*	*No Match*	0.048360473	0	57.318	371.499
TRINITY_DN70519_c4_g1_i1	Protein disulfide-isomerase TMX3	Metazoa; Mammalia; Hominidae	0.048751367	0	16.228	13.761
TRINITY_DN70896_c0_g1_i8	Photoreceptor-specific nuclear receptor	Metazoa; Mammalia; Muridae	0.049148161	0	5.366	26.115
TRINITY_DN65748_c0_g1_i1	*No Match*	*No Match*	0.049279103	0	7.938	97.799

Supplementary Table S3. Gene ontology terms significantly enriched in genes up-regulated in *Malacosteus* relative to *Pachystomias*

Category	Term	Ontology*	FDR
GO:0007601	visual perception	BP	2.54E-21
GO:0050953	sensory perception of light stimulus	BP	2.54E-21
GO:0007600	sensory perception	BP	2.77E-16
GO:0009583	detection of light stimulus	BP	1.99E-13
GO:0050877	neurological system process	BP	2.94E-13
GO:0009416	response to light stimulus	BP	4.48E-10
GO:0007602	phototransduction	BP	1.25E-09
GO:0009581	detection of external stimulus	BP	1.29E-09
GO:0009582	detection of abiotic stimulus	BP	1.33E-09
GO:0009584	detection of visible light	BP	3.31E-09
GO:0051606	detection of stimulus	BP	3.61E-09
GO:0001750	photoreceptor outer segment	CC	3.90E-09
GO:0009314	response to radiation	BP	4.62E-09
GO:0003008	system process	BP	9.11E-09
GO:0097458	neuron part	CC	7.81E-08
GO:0031513	nonmotile primary cilium	CC	9.27E-08
GO:0072372	primary cilium	CC	1.49E-06
GO:0097381	photoreceptor disc membrane	CC	2.02E-06
GO:0007468	regulation of rhodopsin gene expression	BP	5.41E-06
GO:0045872	positive regulation of rhodopsin gene expression	BP	5.41E-06
GO:0060170	ciliary membrane	CC	5.96E-05
GO:0050908	detection of light stimulus involved in visual perception	BP	6.95E-05
GO:0050962	detection of light stimulus involved in sensory perception	BP	6.95E-05
GO:0005215	transporter activity	MF	8.16E-05
GO:0009628	response to abiotic stimulus	BP	8.29E-05
GO:0005929	cilium	CC	0.00012254
GO:0022890	inorganic cation transmembrane transporter activity	MF	0.000146987
GO:0007603	phototransduction, visible light	BP	0.000166326
GO:0001917	photoreceptor inner segment	CC	0.000189183
GO:0060041	retina development in camera-type eye	BP	0.00025176
GO:0050906	detection of stimulus involved in sensory perception	BP	0.000297919
GO:0009605	response to external stimulus	BP	0.000314382
GO:0034703	cation channel complex	CC	0.000692165
GO:0022843	voltage-gated cation channel activity	MF	0.000816449
GO:0015077	monovalent inorganic cation transmembrane transporter activity	MF	0.000991237
GO:0044425	membrane part	CC	0.0010201
GO:0044707	single-multicellular organism process	BP	0.0010201
GO:0032501	multicellular organismal process	BP	0.001080972
GO:0008324	cation transmembrane transporter activity	MF	0.00121961
GO:0006811	ion transport	BP	0.001701798

GO:0046873	metal ion transmembrane transporter activity	MF	0.001763935
GO:0022400	regulation of rhodopsin mediated signaling pathway	BP	0.001999905
GO:0008277	regulation of G-protein coupled receptor protein signaling pathway	BP	0.002156795
GO:0022892	substrate-specific transporter activity	MF	0.002626105
GO:0016056	rhodopsin mediated signaling pathway	BP	0.002659984
GO:0005244	voltage-gated ion channel activity	MF	0.003257917
GO:0022832	voltage-gated channel activity	MF	0.003358873
GO:0005261	cation channel activity	MF	0.004182037
GO:0042706	eye photoreceptor cell fate commitment	BP	0.004182037
GO:0046552	photoreceptor cell fate commitment	BP	0.004182037
GO:0022857	transmembrane transporter activity	MF	0.005779595
GO:0015075	ion transmembrane transporter activity	MF	0.005779595
GO:0016021	integral component of membrane	CC	0.006558404
GO:0005212	structural constituent of eye lens	MF	0.006558404
GO:0022891	substrate-specific transmembrane transporter activity	MF	0.010579876
GO:0031224	intrinsic component of membrane	CC	0.010705711
GO:0030553	cGMP binding	MF	0.010962567
GO:0050896	response to stimulus	BP	0.011415041
GO:0034702	ion channel complex	CC	0.017851406
GO:0005891	voltage-gated calcium channel complex	CC	0.017851406
GO:0008137	NADH dehydrogenase (ubiquinone) activity	MF	0.0198659
GO:0050136	NADH dehydrogenase (quinone) activity	MF	0.0198659
GO:0003954	NADH dehydrogenase activity	MF	0.021434035
GO:0044459	plasma membrane part	CC	0.022955546
GO:0070469	respiratory chain	CC	0.022972051
GO:0016655	oxidoreductase activity, acting on NAD(P)H, quinone or similar compound as acceptor	MF	0.022972051
GO:1902495	transmembrane transporter complex	CC	0.030309848
GO:0055085	transmembrane transport	BP	0.030309848
GO:0005249	voltage-gated potassium channel activity	MF	0.030309848
GO:0071482	cellular response to light stimulus	BP	0.030309848
GO:1990351	transporter complex	CC	0.031483852
GO:0005216	ion channel activity	MF	0.033780214
GO:0001895	retina homeostasis	BP	0.034978258
GO:0015079	potassium ion transmembrane transporter activity	MF	0.035828015
GO:0022838	substrate-specific channel activity	MF	0.035962758
GO:0034704	calcium channel complex	CC	0.036256495
GO:0015267	channel activity	MF	0.040022515
GO:0022803	passive transmembrane transporter activity	MF	0.040022515
GO:0042462	eye photoreceptor cell development	BP	0.041446858
GO:0050804	modulation of synaptic transmission	BP	0.041446858
GO:0005834	heterotrimeric G-protein complex	CC	0.043328608
GO:0042572	retinol metabolic process	BP	0.049616254

*GO term ontology: CC = Cellular component, MF = Molecular Function, BP = Biological Process

Supplementary Table S4. Taxonomic identity and diversity of assigned reads following normalization of transcript number from the *Malacosteus* and *Pachystomias* retina metatranscriptomes

Assigned Taxonomic Clade*	*Malacosteus* # Transcripts	*Pachystomias* # Transcripts	*Malacosteus* % Total reads	*Pachystomias* % Total reads
Metazoa	2340	1537	75.53	69.58
Gammaproteobacteria	148	131	4.78	5.93
Bacilli	111	117	3.58	5.30
Actinobacteria	72	102	2.32	4.62
Fungi	71	61	2.29	2.76
Streptophyta	49	51	1.58	2.31
Betaproteobacteria	43	32	1.39	1.45
Alphaproteobacteria	42	45	1.36	2.04
Clostridia	41	30	1.32	1.36
Apicomplexa	28	5	0.90	0.23
Bacteroidetes	26	24	0.84	1.09
delta/epsilon subdivisions	18	9	0.58	0.41
Oscillatoriophycideae	11	3	0.36	0.14
Chlorophyta	8	5	0.26	0.23
Spirochaetia	7	6	0.23	0.27
Nostocales	6	0	0.19	0.00
Kinetoplastida	6	0	0.19	0.00
Chlamydiae	5	4	0.16	0.18
Negativicutes	4	1	0.13	0.05
Ciliophora	4	4	0.13	0.18
Stigonematales	3	0	0.10	0.00
Pyrenomonadales	3	1	0.10	0.05
Herpesviridae	3	0	0.10	0.00
uncultured bacterium CSL142	2	3	0.06	0.14
Erysipelotrichia	2	6	0.06	0.27
Fusobacteriia	2	1	0.06	0.05
unclassified Bacteria (miscellaneous)	2	0	0.06	0.00
Halobacteria	2	0	0.06	0.00
Methanomicrobia	2	0	0.06	0.00
Oomycetes	2	0	0.06	0.00
unclassified Mimiviridae	2	0	0.06	0.00
unclassified Retroviridae	2	2	0.06	0.09
synthetic construct	2	1	0.06	0.05
Cloning vector pT2/Gene-Trap	2	0	0.06	0.00
Chlorobi	1	0	0.03	0.00
Verrucomicrobia	1	0	0.03	0.00
Deinococci	1	2	0.03	0.09
uncultured bacterium	1	1	0.03	0.05
uncultured bacterium	1	1	0.03	0.05

122006-I05				
uncultured bacterium 4050020-J15	1	1	0.03	0.05
Thermotogae	1	0	0.03	0.00
Omnitrophica	1	0	0.03	0.00
Chromerida	1	1	0.03	0.05
Perkinsea	1	1	0.03	0.05
Archamoebae	1	0	0.03	0.00
Discosea	1	0	0.03	0.00
Mycetozoa	1	0	0.03	0.00
Diplomonadida	1	0	0.03	0.00
Isochrysidales	1	0	0.03	0.00
Prymnesiales	1	0	0.03	0.00
Bacillariophyta	1	1	0.03	0.05
Eustigmatophyceae	1	1	0.03	0.05
PX clade	1	1	0.03	0.05
Asfivirus	1	0	0.03	0.00
Podoviridae	1	1	0.03	0.05
Lymphocystivirus	1	0	0.03	0.00
Megalocytivirus	1	0	0.03	0.00
unclassified Phycodnaviridae	1	0	0.03	0.00
Chordopoxvirinae	1	0	0.03	0.00
Mononegavirales	1	0	0.03	0.00
unclassified ssRNA positive-strand viruses	1	0	0.03	0.00
Prochlorales	0	1	0.00	0.05
uncultured bacterium FGYC_13M19	0	1	0.00	0.05
Acidobacteria	0	2	0.00	0.09
Planctomycetia	0	2	0.00	0.09
Mollicutes	0	3	0.00	0.14
Parcubacteria	0	1	0.00	0.05
Choanoflagellida	0	1	0.00	0.05
Bangiophyceae	0	1	0.00	0.05
Chlorovirus	0	2	0.00	0.09
Astroviridae	0	1	0.00	0.05
uncultured prokaryote	0	1	0.00	0.05
Cloning vector pLMB51	0	1	0.00	0.05
**Total**	**3098**	**2209**	**100.00**	**100.00**

*Taxonomic clades derived from level 4 of the NCBI taxonomy

Supplementary Table S5. Identity of transcripts of putatively bacterial origin from *Malacosteus* and *Pachystomias* retinae overlapping in gene ontology (GO) term annotation with GO terms directly associated with the search term 'Bacteriochlophyll' (S% = 100) and those recorded as being co-annotated with these search terms in previous studies (S% < 100)

GO Name	GO Term	Aspect*	S%	*Malacosteus*		*Pachystomias*	
				RefSeq	BC	RefSeq	BC
Bacteriochlorophyll binding	GO:0042314	MF	100				
Bacteriochlorophyll c binding	GO:0016169	MF	100				
Bacteriochlorophyll metabolic process	GO:0030493	BP	100				
Bacteriochlorophyll catabolic process	GO:0030495	BP	100				
Bacteriochlorophyll biosynthetic process	GO:0030494	BP	100				
Light-dependent bacteriochlorophyll biosynthetic process	GO:0036069	BP	100				
Light-independent bacteriochlorophyll biosynthetic process	GO:0036070	BP	100				
2-desacetyl-2-hydroxyethyl bacteriochlorophyllide a dehydrogenase activity	GO:0036354	MF	100				
Chlorosome	GO:0046858	CC	100				
B875 antenna complex	GO:0030080	CC	100				
Light-harvesting complex, peripheral complex	GO:0030079	CC	100				
light-harvesting complex	GO:0030076	CC	72.73				
plasma membrane light-harvesting complex	GO:0030077	CC	26.68				
organelle inner membrane	GO:0019866	CC	8.25				
chlorophyll binding	GO:0016168	MF	0.08-5.21				

electron transporter, transferring electrons within the cyclic electron transport pathway of photosynthesis activity	GO:0045156	MF	3.6			
photosynthesis, light reaction	GO:0019684	BP	2.16			
plasma membrane-derived chromatophore membrane	GO:0042717	CC	2.08			
protein-chromophore linkage	GO:0018298	BP	0.03-1.94			
chlorosome envelope	GO:0033105	BP	1.39-76.47			
response to herbicide	GO:0009635	BP	0.16			
photosynthetic electron transport in photosystem II	GO:0009772	BP	0.11			
plasma membrane	GO:0005886	CC	0.05			TRINITY_PA_BC71_c0_g1_i1
photosynthesis	GO:0015979	BP	0.01-1.14			
metal ion binding	GO:0046872	MF	0.02-0.03	TRINITY_MA_RF190_c0_g1_i1<br>TRINITY_MA_RF108_c0_g2_i1<br>TRINITY_MA_RF108_c0_g1_i1		TRINITY_PA_BC71_c0_g1_i1
integral component of membrane	GO:0016021	CC	0.01			
membrane	GO:0016020	CC	0.01		TRINITY_PA_RF133_c0_g1_i1	TRINITY_PA_BC71_c0_g1_i1
oxidoreductase activity, acting on iron-sulfur proteins as donors	GO:0016730	MF	18.00-22.57			
photosynthesis, dark reaction	GO:0019685	BP	22.27-24.15			
pigment biosynthetic process	GO:0046148	BP	15.57-21.63			
oxidoreductase activity, acting on the CH-CH group of donors, iron-sulfur protein as acceptor	GO:0016636	MF	14.67			
chlorophyll biosynthetic process	GO:0015995	BP	0.03-13.96			
oxidoreductase activity, acting on iron-sulfur proteins as donors, NAD or NADP as acceptor	GO:0016731	MF	0.11-7.21			

magnesium chelatase activity	GO:0016851	MF	0.12-6.16	
magnesium-protoporphyrin IX monomethyl ester (oxidative) cyclase activity	GO:0048529	MF	0.07-12.35	
oxidoreductase activity, acting on the CH-CH group of donors, NAD or NADP as acceptor	GO:0016628	MF	0.01-22.43	
carbonyl sulfide nitrogenase activity	GO:0018697	MF	0.82	
hydro-lyase activity	GO:0016836	MF	0.01-0.65	
nitrogenase activity	GO:0016163	MF	0.57	
protochlorophyllide reductase activity	GO:0016630	MF	0.55	
4 iron, 4 sulfur cluster binding	GO:0051539	BP	0.19-0.25	
porphyrin-containing compound biosynthetic process	GO:0006779	BP	0.22	
iron-sulfur cluster binding	GO:0051536	MF	0.11-0.15	
oxidoreductase activity	GO:0016491	MF	0.01-0.04	TRINITY_PA_RF202_c0_g1_i1<br>TRINITY_PA_RF164_c0_g1_i<br>TRINITY_PA_RF77_c0_g1_i1<br>TRINITY_PA_RF164_c1_g1_i1<br>TRINITY_PA_RF226_c0_g1_i1<br>TRINITY_PA_RF34_c0_g1_i1
divinyl chlorophyllide a 8-vinyl-reductase activity	GO:0033728	MF	0.04	
D-xylulose reductase activity	GO:0046526	MF	0.04-0.25	
L-threonine 3-dehydrogenase activity	GO:0008743	MF	0.04-0.06	

oxidation-reduction process	GO:0055114	BP	0.02-0.04			TRINITY_PA_RF164_c0_g1_i1<br>TRINITY_PA_RF202_c0_g1_i1<br>TRINITY_PA_RF77_c0_g1_i<br>TRINITY_PA_RF164_c1_g1_i1<br>TRINITY_PA_RF226_c0_g1_i1<br>TRINITY_PA_RF34_c0_g1_i1	TRINITY_PA_BC71_c0_g1_i1
magnesium protoporphyrin IX methyltransferase activity	GO:0046406	MF	0.03				
ligase activity	GO:0016874	MF	0.03				
nucleotide binding	GO:0000166	MF	0.02-0.03	TRINITY_MA_RF53_c0_g1_i1<br>TRINITY_MA_RF81_c0_g1_i1<br>TRINITY_MA_RF150_c1_g1_i1<br>TRINITY_MA_RF150_c0_g1_i1<br>TRINITY_MA_RF139_c0_g1_i1<br>TRINITY_MA_RF3_c0_g1_i1	TRINITY_MA_BC9_c0_g1_i1	TRINITY_PA_RF207_c0_g1_i1<br>TRINITY_PA_RF133_c0_g1_i1<br>TRINITY_PA_RF201_c0_g1_i1<br>TRINITY_PA_RF35_c0_g1_i1<br>TRINITY_PA_RF77_c0_g1_i1<br>TRINITY_PA_RF226_c0_g1_i1	TRINITY_PA_BC69_c0_g1_i1
geranylgeranyl reductase activity	GO:0045550	MF	0.03-0.06				
ATP binding	GO:0005524	MF	0.02-0.03	TRINITY_MA_RF53_c0_g1_i1<br>TRINITY_MA_RF150_c1_g1_i1<br>TRINITY_MA_RF150_c0_g1_i1	TRINITY_MA_BC9_c0_g1_i1		
coproporphyrinogen dehydrogenase activity	GO:0051989	MF	0.02				
oxidoreductase activity, acting on paired donors, with incorporation or reduction of molecular oxygen, NAD(P)H as one donor, and incorporation of one atom of oxygen	GO:0016709	MF	0.02				
iron ion binding	GO:0005506	MF	0.02				
zinc ion binding	GO:0008270	MF	0.02				
coproporphyrinogen oxidase activity	GO:0004109	MF	0.01				
sigma factor activity	GO:0016987	MF	0.01				

DNA-templated transcription, initiation	GO:0006352	BP	0.01	TRINITY_PA_BC71_c0_g1_i1
cobalamin binding	GO:0031419	MF	0.01	
prenyltransferase activity	GO:0004659	MF	0.01	

*GO term ontology: CC = Cellular component, MF = Molecular Function, BP = Biological Process
